# Inhibitory Effect of Curcumin-Inspired Derivatives on Tyrosinase Activity and Melanogenesis

**DOI:** 10.3390/molecules27227942

**Published:** 2022-11-16

**Authors:** Gaia Rocchitta, Carla Rozzo, Marina Pisano, Davide Fabbri, Maria Antonietta Dettori, Paolo Ruzza, Claudia Honisch, Roberto Dallocchio, Alessandro Dessì, Rossana Migheli, PierAndrea Serra, Giovanna Delogu

**Affiliations:** 1 Dipartimento di Medicina, Chirurgia e Farmacia, Università Degli Studi di Sassari, 07100 Sassari, Italy; 2 Istituto di Ricerca Genetica e Biomedica, Consiglio Nazionale Ricerche, 07100 Sassari, Italy; 3 Istituto di Chimica Biomolecolare, Consiglio Nazionale Ricerche, 07100 Sassari, Italy; 4 Istituto di Chimica Biomolecolare, Consiglio Nazionale Ricerche, 35131 Padova, Italy

**Keywords:** tyrosinase inhibitors, sustainable synthesis, curcumin-inspired derivatives, melanogenesis, in silico analyses, biosensors, antioxidant activity, hyperpigmentation

## Abstract

Tyrosinase is a well-known copper-containing metalloenzyme typically involved in the synthesis of melanin. Recently, curcumin and several synthetic derivatives have been recognized as tyrosinase inhibitors with interesting anti-melanogenic therapeutic activity. In this study, three curcumin-inspired compounds **1**, **6** and **7** were prepared in yields ranging from 60 to 88 % and spectrophotometric, electrochemical, in vitro and in silico analyses were carried out. The viability of PC12 cells, a rat pheochromocytoma derived-cell line, with compounds **1**, **6** and **7**, showed values around 80% at 5 µM concentration. In cell proliferation assays, compounds **1**, **6** and **7** did not show significant toxicity on fibroblasts nor melanoma cells up to 10 µM with viability values over 90%. The inhibition of tyrosinase activity was evaluated both by a UV-Vis spectroscopic method at two different concentrations, 0.2 and 2.0 µM, and by amperometric assay with IC_50_ for compounds **1**, **6** and **7** ranging from 11 to 24 nM. Melanin content assays on human melanoma cells were performed to test the capability of compounds to inhibit melanin biosynthesis. All compounds exerted a decrease in melanin content, with compound **7** being the most effective by showing a melanogenesis inhibition up to four times greater than arbutin at 100 µM. Moreover, the antioxidant activity of the selected inhibitors was evaluated against H_2_O_2_ in amperometric experiments, whereby compound **7** was about three times more effective compared to compounds **1** and **6**. The tyrosinase X-ray structure of *Bacterium megaterium* crystal was used to carry out molecular docking studies in the presence of compounds **1**, **6** and **7** in comparison with that of kojic acid and arbutin, two conventional tyrosinase inhibitors. Molecular docking of compounds **6** and **7** confirmed the high affinity of these compounds to tyrosinase protein.

## 1. Introduction

**Figure 1:**
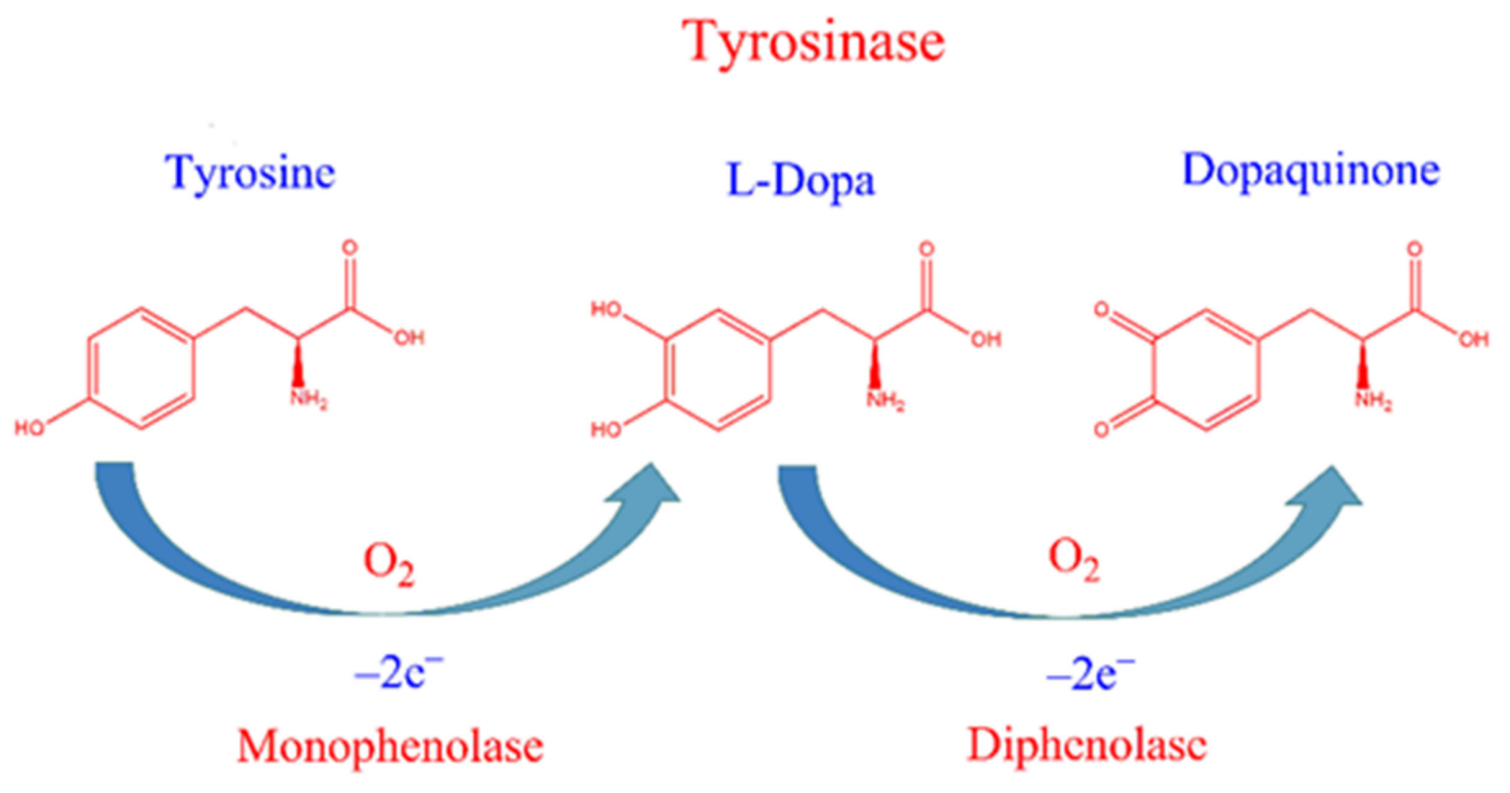
Tyrosinase enzyme reactions.

Melanin is the main pigment responsible for the color of human skin, eyes, and hair [1]. It is produced mainly around the nucleus of melanocytes and stored in melanosomes. Melanin protects the skin from ultraviolet irradiation that induces oxidative stress producing wrinkling and DNA damage. However, overproduction or accumulation of melanin provokes hyperpigmentation, dermatological disorders, freckles, solar lentigines and age spots [2]. Tyrosinase (E.C. 1.14.18.1) is a copper-containing metalloenzyme that catalyzes the initial steps of melanin biosynthesis through two successive activities: monophenolase and diphenolase cycles [3,4]. Two copper ions catalyze a sequence of redox reactions, the first cycle of reactions consists in the hydroxylation of monophenol *L*-tyrosine, natural subtract of tyrosinase, to *o*-diphenol (L-DOPA), whereas the second one involves oxidation of the *o*-diphenol to the corresponding *o*-quinone (dopaquinone) (Figure 1).

Both reactions are coupled with the reduction of dioxygen to water. The resulting oxidative intermediates produce, after polymerization reactions, pheomelanin and eumelanin, two pigments forming melanin [5]. The catalytic mechanism of tyrosinase is known to involve several different states. In the monophenolase activity (oxidation of L-tyrosine to L-DOPA), the enzyme goes through four different isoforms (E_deoxy_, E_oxy_, E_oxy-M_, and E_met-D_), while during the oxidation of L-DOPA to dopaquinone (diphenolase or cathecolase activity), the enzyme passes through five states (E_deoxy_, E_oxy_, E_oxy-D_, E_met_, and E_met-D_) related to the different oxidation states of copper ions [3].

Since tyrosinase is the key rate-limiting enzyme in melanin biosynthesis, this enzyme can be addressed as the biological target to design skin whitening and anti-melanogenic agents [6].

**Figure 2:**
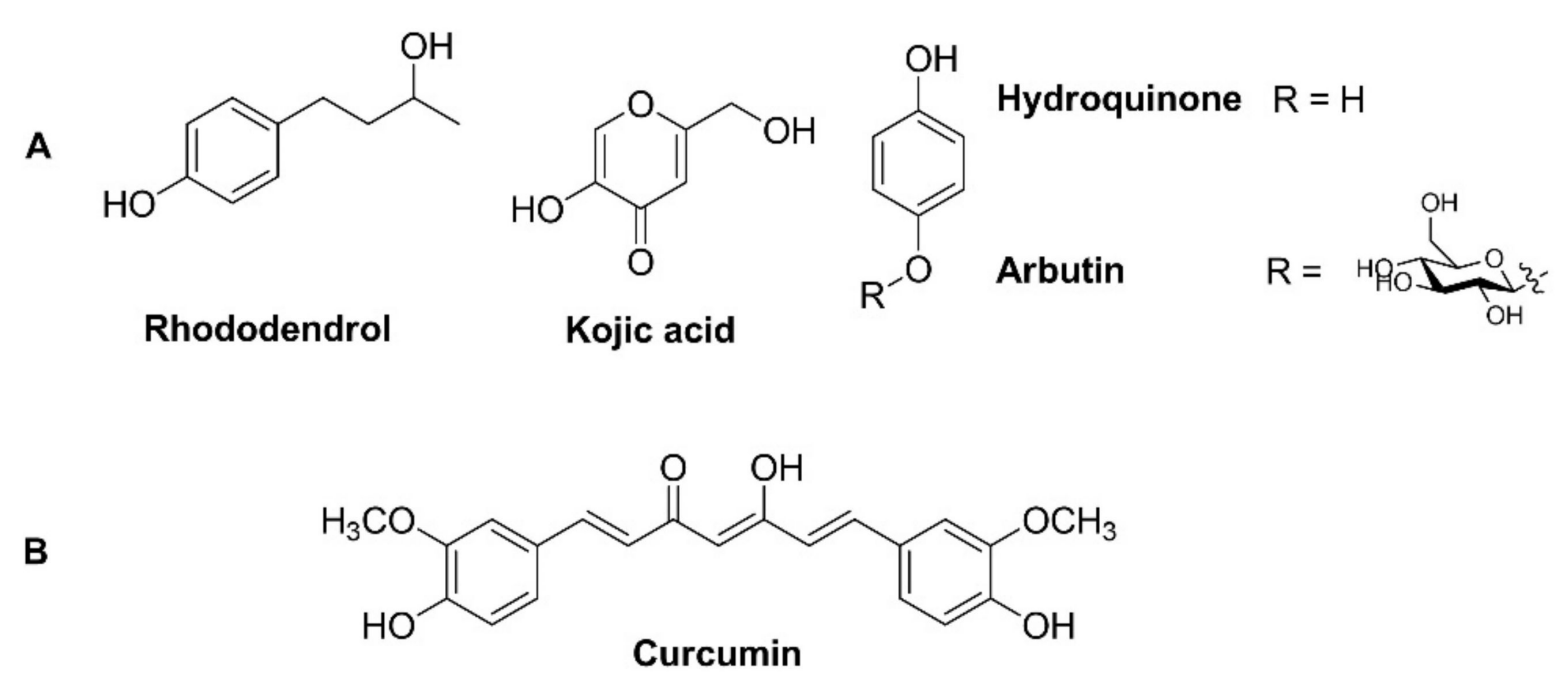
(**A**) Natural tyrosinase inhibitors (**B**) Curcumin structure.

For years, only a few naturally occurring compounds, including arbutin, kojic acid and rhododendrol (Figure 2A), have been used as cosmetic additives since most of the proposed melanogenesis inhibitors and skin whitening agents have generated concern due to their toxicity, scarce membrane crossing, or low inhibitory activity [7,8,9,10,11].

Recently, several nature-inspired tyrosinase inhibitors have been prepared and proposed as an alternative to those commercially available [12,13,14,15,16]. The crystal structure of *Bacterium megaterium* tyrosinase, containing or not one molecule of kojic acid, was obtained at high resolution [17,18], allowing to shed some light on the mechanism of the inhibitor/substrate reaction since the catalytic site of tyrosinase is well-conserved in human, animals, bacteria, fungi and plants [19]. Recently, curcumin (Figure 2B) and several synthetic derivatives have been recognized as tyrosinase inhibitors with interesting anti-melanogenic activity [20,21,22]. Curcumin (*Curcuma longa*) is one of the most common spicy foods and flavoring agents and is generally recognized as safe and effective against a plethora of diseases and neurodegenerative disorders [23,24]. Analyzing the structural motif of curcumin-inspired tyrosinase inhibitors, the hydroxylated phenol unit bonded to an aliphatic chain containing a β diketone moiety appears to be essential for the activity of the molecule [25,26]. Hence, it is necessary to develop more effective and safer tyrosinase inhibitors in order to design skin whitening and anti-melanogenic agents with great market potential.

**Figure 3:**
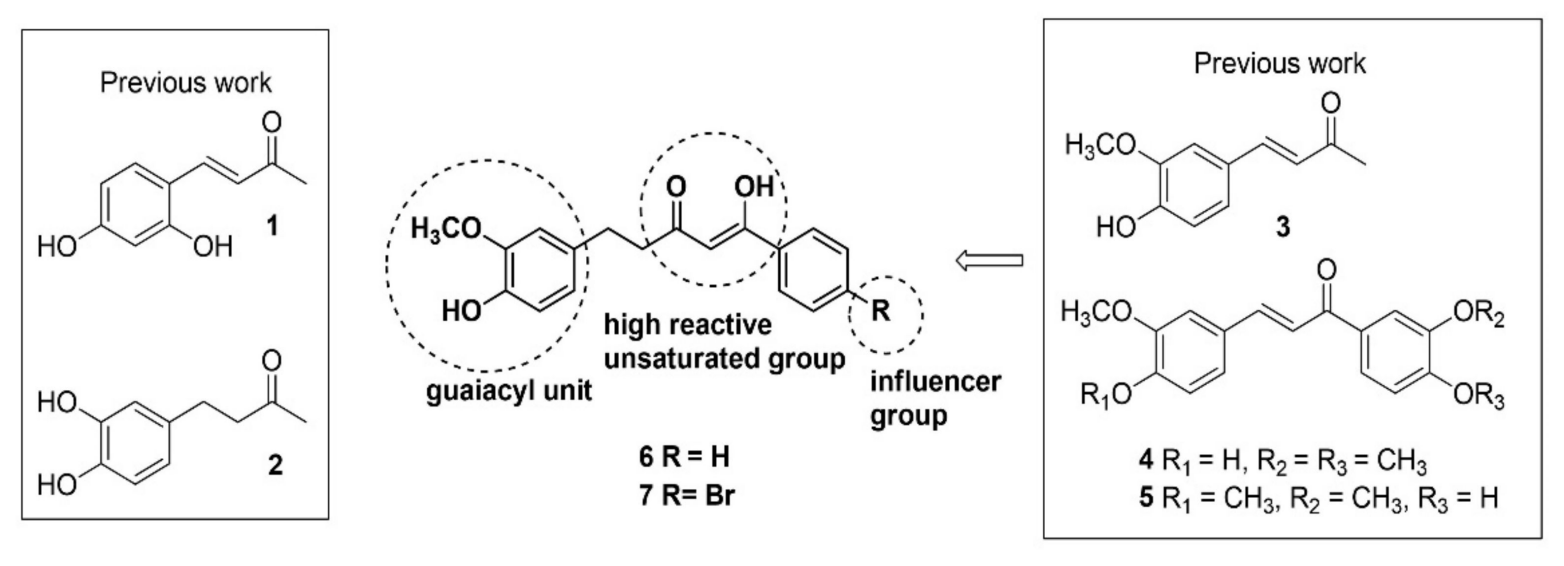
Design of tyrosinase inhibitors **6** and **7** based on the chemical structure of inhibitors **3**–**5**.

Recently we prepared a small library of hydroxylated phenylpropanoids, compounds **1**–**5** (Figure 3), and we determined competitive and mixed tyrosinase inhibition by biosensor detection and IC_50_ concentration ranging from 20 to 423 nM [27]. This work aimed to enhance tyrosinase inhibition and to decrease the cytotoxicity of the compounds for an application both in melanogenesis disorders and skin whiteners. For the synthesis of compounds **6** and **7**, we decided to maintain the guaiacyl core, a recognized safe pharmacophore present in molecules **3**–**5,** and to modify the geometrical feature of the aliphatic chain by introducing a 1,3-diketo function, susceptible to electron transfer reactions with the amino acid residues of the tyrosinase catalytic site (Figure 3). Compound **1** is a potent tyrosinase inhibitor, as detected in both spectrophotometric and electrochemical assays, in accordance with the literature data [26,27]. This compound was used in this work as a control. A comparative study on spectrophotometric, electrochemical, in vivo and in silico analyses was carried out.

## 2. Results

### 2.1. Synthesis of Inhibitors

**Scheme 1:**
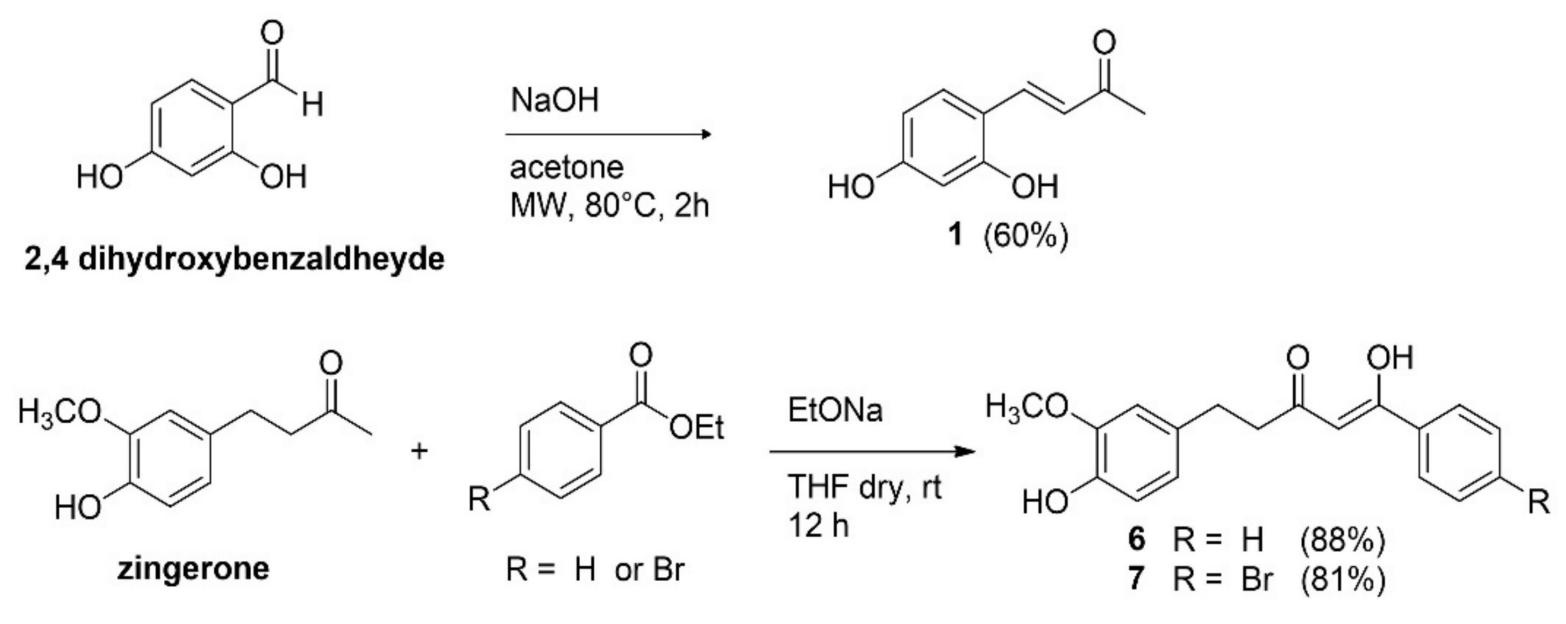
Synthesis of compounds **1**, **6** and **7**.

Compound **1** was prepared with the same yield and purity according to the method previously described by our group [27] but with a more sustainable procedure, carrying out the reaction under microwave irradiation, and reducing the reaction time from 12 h to 2 h. Compounds **6** and **7** were prepared by Claisen condensation of zingerone, a naturally occurring compound, in the presence of the corresponding aryl acetate under basic conditions (Scheme 1) [28].

A nucleophilic attack of the deprotonated base generated in the methyl ketone by sodium ethoxide to the carboxylic carbon of the ester and further loss of the ethoxy group and water gave compounds **6** and **7**. The keto-enol function was confirmed by ^1^H-NMR spectra. The synthetic procedures applied in this work for compounds **6** and **7** were more straightforward and sustainable than those used in the literature [29].

### 2.2. Measurement of Tyrosinase Inhibitory Activity

#### 2.2.1. Mushroom Tyrosinase Spectrophotometric Assay

The capability of molecules to inhibit the activity of tyrosinase has been estimated first by a UV-Vis spectroscopic method evaluating the changes in the UV-Vis spectrum of two natural substrates, L-Tyr and dopamine [27,30]. The near-UV spectrum of L-Tyr in buffer solution before the addition of enzyme is characterized by the presence of a strong band at about 275 nm. The addition of the enzyme firstly hydroxylated the Tyr residue to dihydroxyphenylalanine (DOPA) and successively oxidized DOPA to dopaquinone, which was then converted to dopachrome. Both DOPA and dopaquinone appearances are detected by the change in the UV-Vis spectrum. The hydroxylation of Tyr to DOPA is characterized by an increase in the intensity of the band at 275 nm and the appearance of an additional band at about 304 nm, while the oxidation to dopaquinone is detected by the appearance of a band at 470–490 nm (Figure S1 in Supplementary Materials). The near-UV spectrum of dopamine showed a strong absorption band at about 300 nm and the addition of tyrosinase induced the appearance of a band at about 470 nm due to the oxidation of dopamine to the corresponding quinone (Figure S2 in Supplementary Materials).

The time course of tyrosine oxidation, obtained by monitoring the absorbance at either 477 or 304 nm, showed a characteristic lag-phase due to the low amount of the *oxy* form of the tyrosinase enzyme in the commercially available preparation involved in the first step of tyrosine hydroxylation. Among the different factors that influence the presence and the length of this phase, the presence of catechols induces its disappearance. Indeed, the time course of dopamine oxidation obtained by monitoring the appearance of the absorption band due to the quinone is devoid of this lag phase (Figures S1 and S2 in Supplementary Materials).

**Figure 4:**
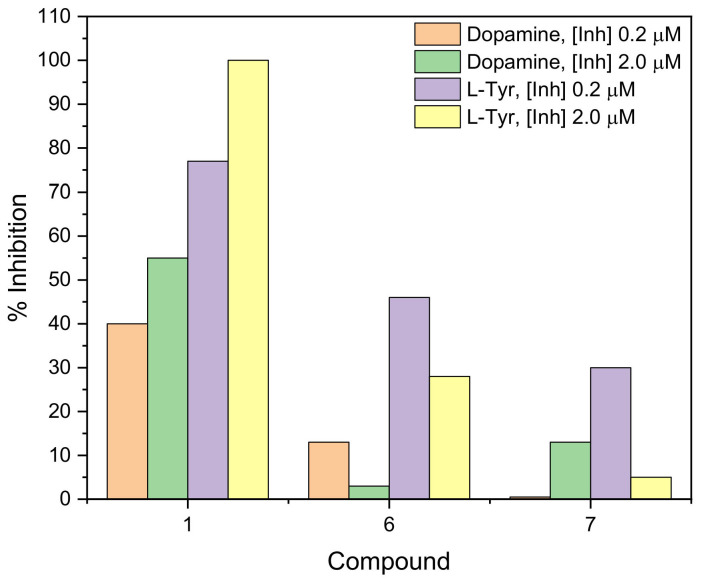
Percentage of tyrosinase inhibition determined by UV-Vis spectroscopy using either L-Tyrosine or dopamine at 0.2 and 2.0 µM as substrate.

The capability of compounds **1**, **6**, and **7** to inhibit the tyrosinase activity was evaluated at two different concentrations, 0.2 and 2.0 µM, and the determined inhibition percentage is reported in Figure 4.

The addition of compound **1** strongly decreased the tyrosinase activity, especially in the presence of L-Tyr as a substrate. On the contrary, compounds **6** and **7** appeared to be ineffective inhibitors of the tyrosinase enzyme, especially in the presence of dopamine as a substrate.

#### 2.2.2. Mushroom Tyrosinase Biosensor Assay

The tyrosinase inhibition activity of molecules **1**, **6** and **7** has been estimated also by amperometric enzyme-based biosensors. The kinetic performances of tyrosinase-based biosensors, the type of inhibitions and IC_50_ values are reported in Table 1 and in Figures S4–S6 in the Supplementary Materials.

Data were obtained for each studied compound. As highlighted, only compound **7** determined a significative (*p* < 0.05 vs. dopamine) variation in V_MAX_ of about −28%, while all compounds produced significative (*p* < 0.05 vs. dopamine) increases in K_M_ values. So, in case of compounds **1** and **6**, a competitive inhibition was assessed, while for compound **7** a mixed inhibition was reported. The calculated IC_50_ values were comparable for compounds **6** and **7** (11 nM and 12 nM, respectively), while for compound **1** the value was 24 nM in line with the previously published value [27].

### 2.3. Antioxidant Activity of the Compounds via Amperometric Assay

As previously described [31], when H_2_O_2_ and a reducing compound are present simultaneously, it is possible that a homogeneous reaction occurs in the electrochemical cell. So, the electrodes were properly modified through the electrodeposition of a poly-*o*-phenylenediamine (PPD) polymer, allowing the surface of the electrode to detect only the currents derived from the reduction in H_2_O_2_. Monitoring the H_2_O_2_ current alone permitted us to estimate the current modifications in the presence of increasing concentrations of reducing species (with antioxidant activity).

**Figure 5:**
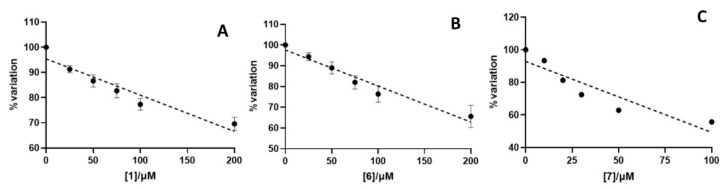
Antioxidant activity exerted on a fixed concentration of H_2_O_2_ (200 µM) by compound **1** (**A**), **6** (**B**) or **7** (**C**) in a range comprised between 0 and 200 µM, dependent on the solubility of compounds.

As shown in Figure 5, while compound **1** produced a decrease in H_2_O_2_ currents of about 0.144% for each µM added in the electrochemical cell, compound **6** produced a reduction of 0.174% for each µM, while compound **7** reduced the H_2_O_2_ current by 0.436% for µM instead. As shown in Figure 5A, compound **1** lessened the current of H_2_O_2_ in a dose-dependent manner, up to 200 µM, with a decrease of about 28.5%. A similar trend was observed with compounds **6** and **7** (Figure 5B,C), which determined a slightly higher decrease if compared to **1** of about 36.6% and 44.2%, respectively.

Compound **1** presented a slope of −0.216 ± 0.034 nA/µM (R^2^ = 0.910), **6** showed instead a slope equal to −0.228 ± 0.018 nA/µM (R^2^ = 0.882), while compound **7** displayed a slope of −0.805 ± 0.075 nA/µM (R^2^ = 0.841), demonstrating an antioxidant activity against H_2_O_2_ about 3.6 times higher than the other molecules.

### 2.4. Cell Culture

#### 2.4.1. Toxicity of Compounds on Melanoma Cells and Fibroblasts

Cell proliferation assays were performed to test the possible cytotoxicity of the compounds. Two human melanoma cell lines (LCP-mel and PNP-mel) and mouse melanoma cell line B16F10, together with a human normal fibroblasts cell line BJ, were grown up to 48 h each in the presence of compounds **1**, **6**, or **7**, at increasing concentrations of 1, 10 and 100 µM. As a positive control we used 10 µM cisplatin, a chemotherapeutic agent known to be a potent cytotoxic drug on these cell lines.

**Figure 6:**
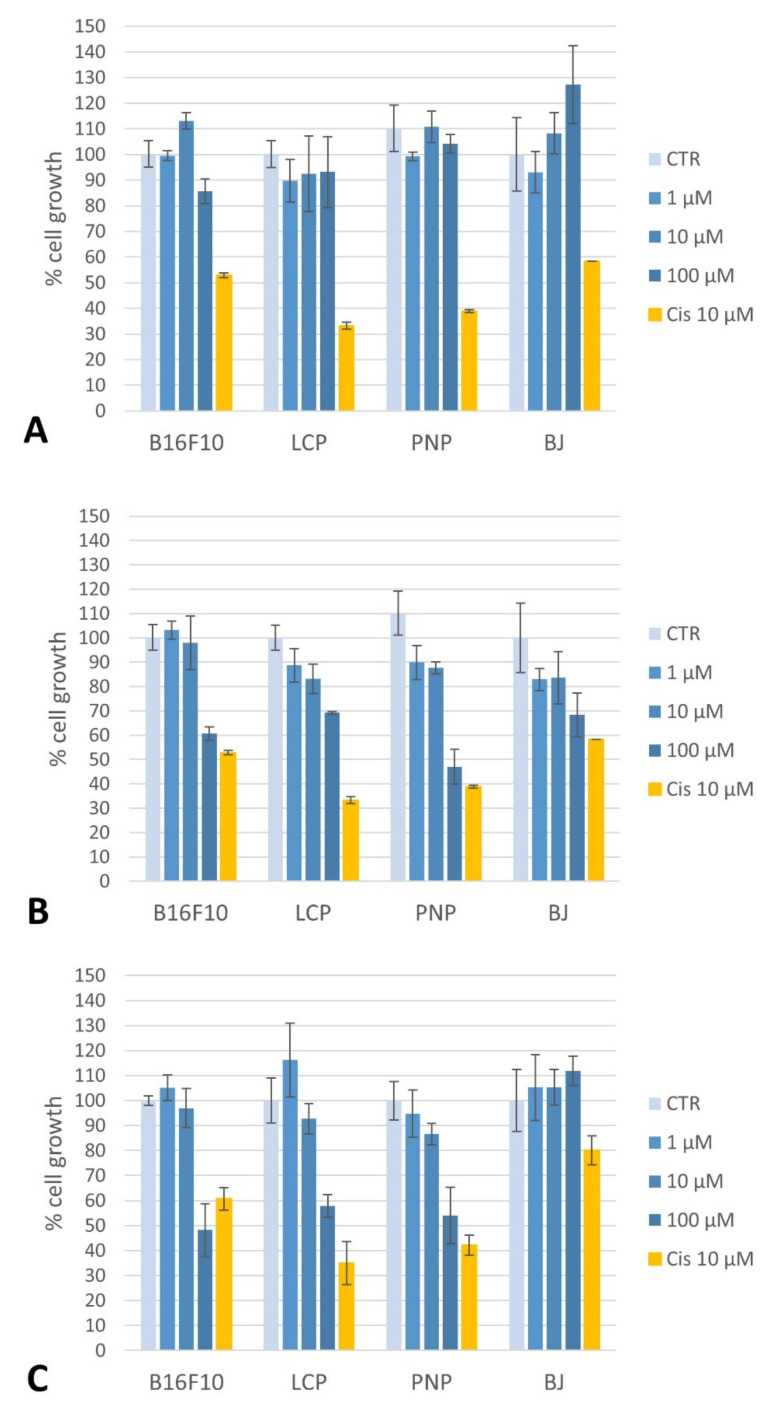
Cytotoxic activity: cells were cultured with increasing concentrations (1, 10, 100 µM) of compounds **1** (**A**), **6** (**B**) or **7** (**C**) up to 48 h. 10 µM Cisplatin (Cis) was used as toxicity positive control. Cell proliferation values were calculated as growth percentages of treated cells compared to the untreated ones (CTR). Graphs represent the results of three experiments, each done in triplicate.

Our results (Figure 6) show that the tyrosinase inhibitor **1,** as well as **6** and **7,** when used at a concentration lower than 100 µM, did not exert significant toxicity on the tested cell lines. In particular, compound **1** was not toxic to any of them at concentrations up to 100 µM. This behavior is comparable to that of arbutin, a known tyrosinase inhibitor currently used in cosmesis as a whitener agent and used as the control in our experiments (Figure S3 in Supplementary Material). Compounds **6** and **7** instead, at 100 µM concentration, were shown to decrease cell growth to 50% on certain cell lines (i.e., on PNP melanoma cells), showing to be slightly toxic if used at this high concentration. Compound **7**, however, showed a little toxicity on melanoma cells but was not toxic to fibroblast, even at 100 µM.

#### 2.4.2. Inhibition of Melanogenesis

To test the capability of our compounds to inhibit melanin synthesis we performed melanin content assays on LCP-mel and PNP-mel human melanoma cells and B16F10 mouse melanoma cells. These cell lines constitutively produce a small amount of melanin, but to better highlight the putative inhibitory effect of our compounds, melanin synthesis was strongly induced in these cells by pre-treating them with a melanocyte stimulating hormone (α-MSH), as described in the Materials and Methods.

Cells were treated with 100 µM of either **1**, **6** or **7** and, in parallel, with 250 µM of arbutin, in order to compare the inhibitory effect of our compounds with that of a known skin whitener agent.

**Figure 7:**
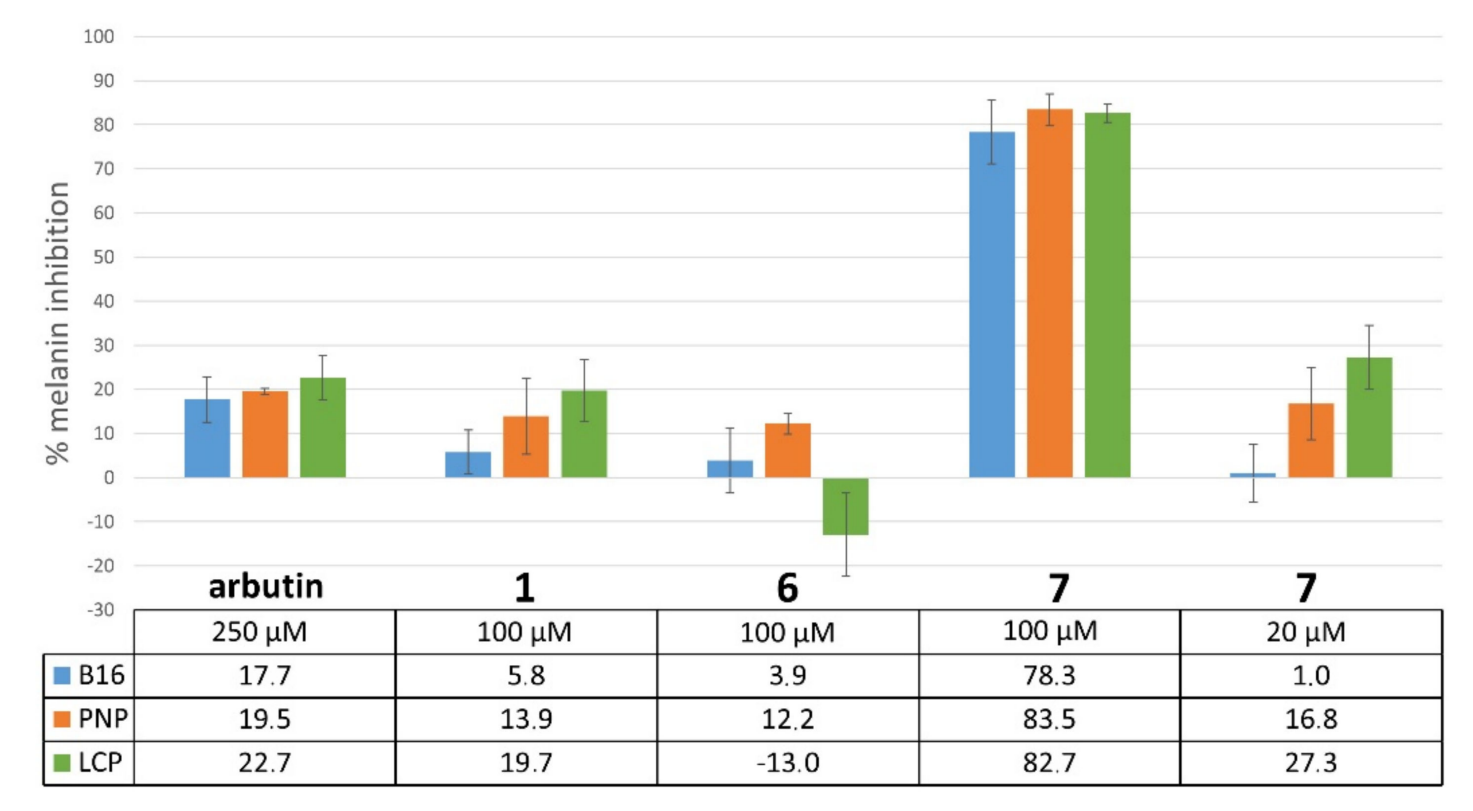
Inhibition of melanogenesis: B16F10 mouse melanoma cells (B16), PNP—mel (PNP) and LCP—mel (LCP) human melanoma cells, stimulated with 10 nM αMSH, where grown in the presence of 250 µM arbutin or 100 µM of either **1, 6** or **7** and 20 µM of **7**, up to 48 h, as described in Materials and Methods. Percentages of melanin inhibition were calculated by comparing melanin content of αMSH—stimulated-treated cells to that of αMSH—stimulated-untreated cells (0% of inhibition). Data represent the mean results obtained by three experiments, each done in triplicate.

As shown in Figure 7, all three compounds caused a decrease in melanin content, especially in human melanoma cells, with compound **7** strongly active at 100 µM (mean 82.8 ± 2.8 % of melanin inhibition). Due to the strong inhibitory activity of compound **7** and knowing its partial toxicity at 100 µM concentration (Figure 6), a lower concentration (20 µM) was also used for this compound to test its efficacy at low doses. Even in this case, a discrete inhibition of melanin content was observed comparable to that of arbutin, which however was used at a dose more than ten times higher (Figure 7). Compound **7** shows then a melanin inhibitor effect comparable to that of arbutin on human melanoma cells, but it is active at much lower concentrations (20 µM vs. 250 µM).

#### 2.4.3. Toxicity of Compounds on PC12 Cells and Protective Activity against Oxidative Stress

Increasing concentrations of the investigated inhibitors were also screened on PC12 cells in order to further evaluate their possible cytotoxicity [32]. As shown in Figure S7 in the Supplementary Materials, compounds **6** and **7** (panel B and C, respectively) were found to be non-toxic towards PC12 cells at all tested concentrations (1–40 μM), as they maintained the cell viability around 80%, albeit causing a significant decrease (*p* < 0.05) if compared to the control. On the contrary, compound **1** (Figure S7, panel A) produced a significant decrease in viability (*p* < 0.05) if compared to the control, showing slight toxicity towards cell cultures starting from a concentration of 10 μM.

Once the toxicity of these compounds was clarified, their protective activity against oxidative stress H_2_O_2_ was assessed.

**Figure 8:**
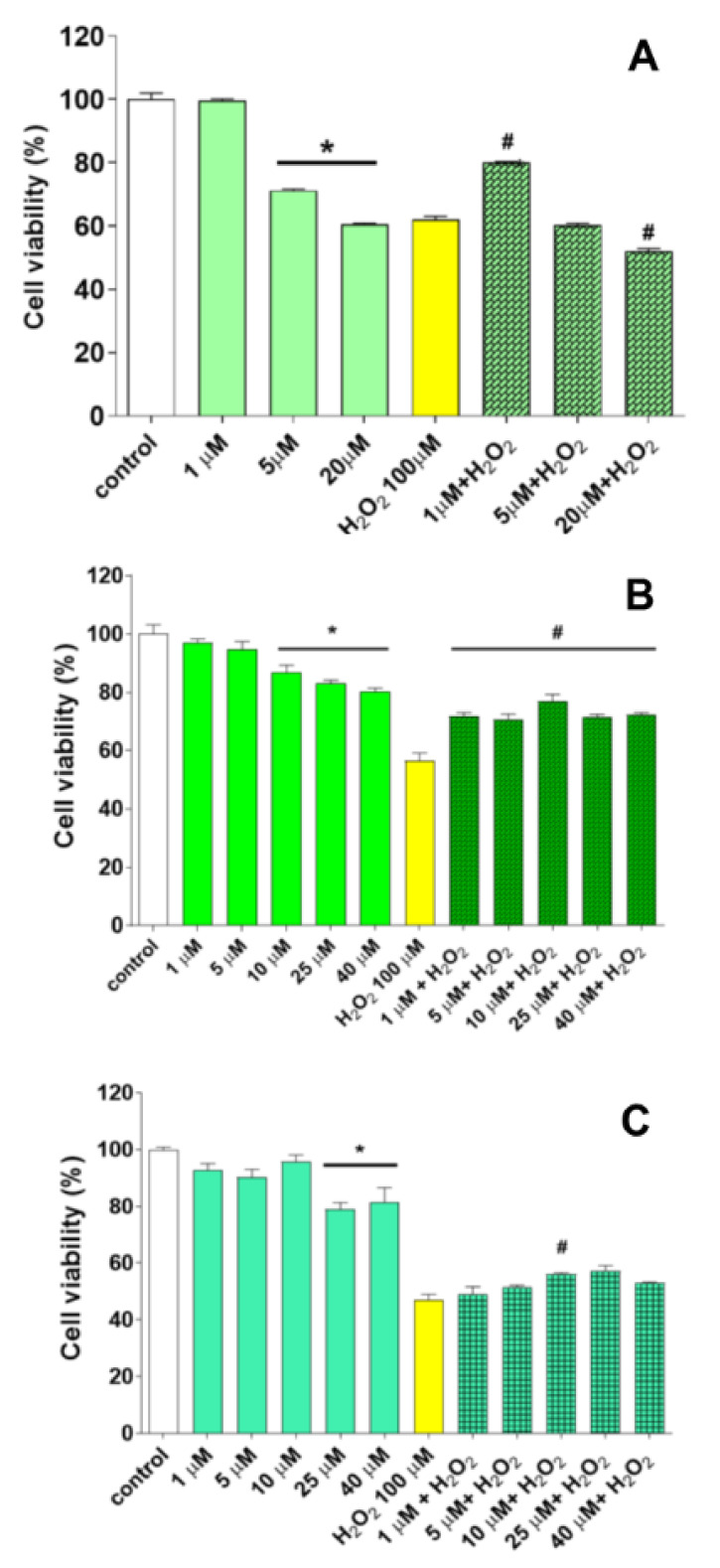
Effects of compounds **1** (**A**), **6** (**B**) and **7** (**C**) on damage induced by H_2_O_2_ (100 µM). Experiments were made on PC12 cells. MTT assay, to assess cell viability, was performed 24 h after each treatment. * *p* < 0.05 vs. control; # *p* < 0.05 vs. H_2_O_2_. In each panel: white column: control viability; no-texture columns: viability associated with increasing concentrations of the compound; yellow column: 100 µM H_2_O_2_ associated viability; textured columns: viability associated with increasing concentrations of the compound in the presence of 100 µM H_2_O_2_.

As depicted in Figure 8, compound **1** was able to restore H_2_O_2_ damage at 1 µM concentration, while compound **7** at 10 µM concentration. On the contrary, compound **6** was able to counteract the damage induced by H_2_O_2_ throughout the range of tested concentrations (1–40 µM).

### 2.5. Molecular Docking Studies

The tyrosinase X-ray structure of *B. megaterium* tyrosinase [PDB file: 3NM8] [17] was used to carry out molecular docking studies with compounds **1**, **6** and **7** and with kojic acid and arbutin, two conventional tyrosinase inhibitors. The active site of tyrosinase consists of two copper ions. Each metal activates strong interactions with a set of three histidine residues: His42, His60 and His69, which bind with one copper ion, and His204, His208 and His231, which interact with the other one.

**Figure 9:**
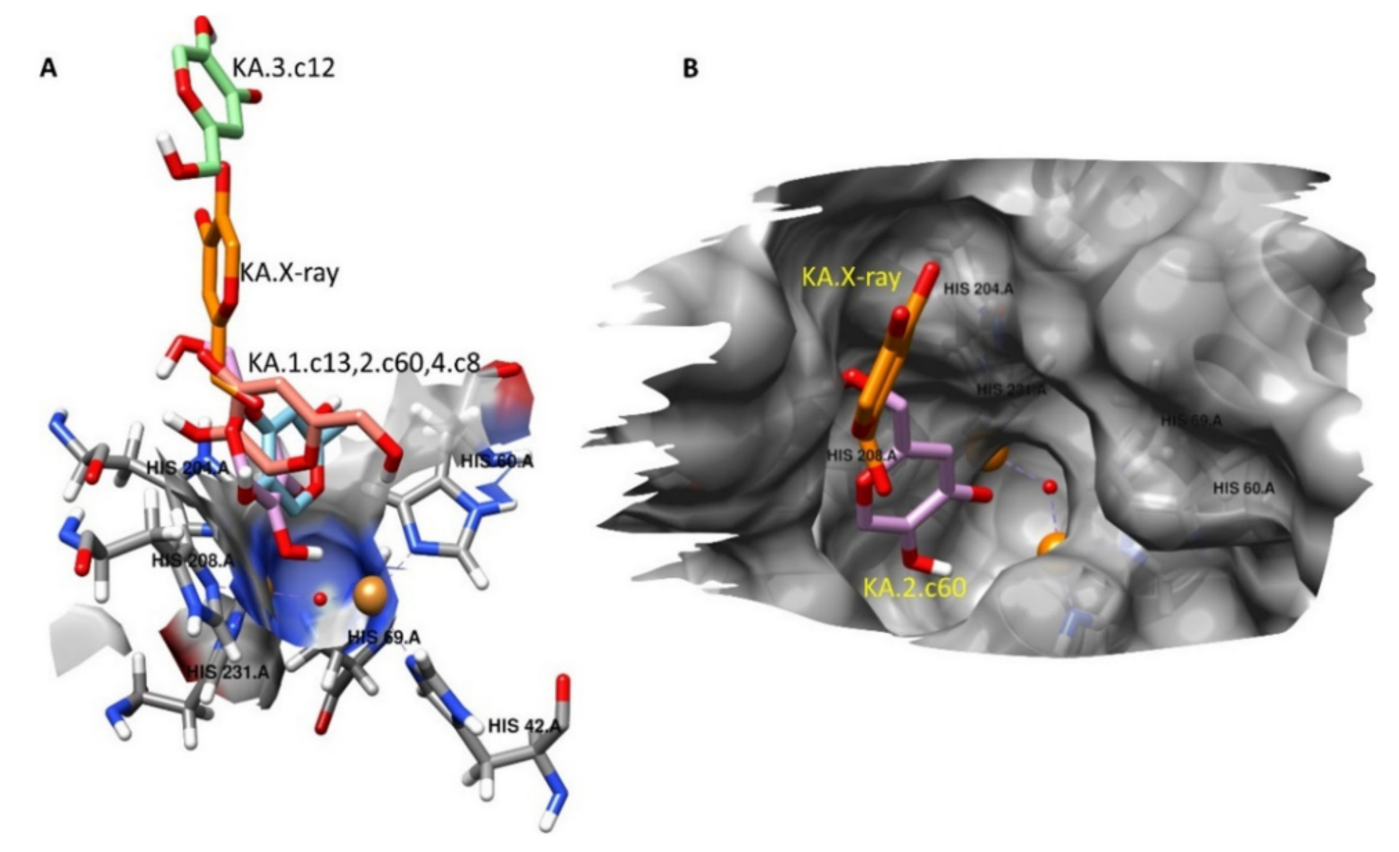
Representation of the catalytic site of *B. megaterium* tyrosinase (crystal 3NQ1): (**A**) Copper ions (orange balls), kojic acid (KA) in X-ray (orange), the best docking poses of kojic acid (magenta, blue and pink), the six histidines (turquois) coordinated to the copper ions. The red sphere represents a molecule of water. (**B**) Entrance of the catalytic site. Kojic acid in the X-ray structure (orange) and the best scoring position (violet). Copper ions (orange balls) and molecule of water (red ball) [17].

In the crystal structure containing kojic acid [PDB file: 3QN1] [17], the acid is positioned at the entrance of the catalytic site, 7 Å away from the copper center. Kojic acid activates strong interactions with amino acids residues Phe197, Pro201, Asn205 and Arg209 that characterize the boundary of a channel facing the catalytic site and stabilize the entrance of the acid (Figure 9) [17,18].

In this representation, kojic acid does not activate strong interactions with the molecule of water bound to copper ions because the acid is positioned in proximity to the entrance of the active site. On the contrary, the best poses of kojic acid (roughly 73%) obtained in the molecular docking with crystal structure 3NM8 evidenced strong interactions (hydrophobic and H-bond) with His204, Asn205, His208, Arg209 and Gly216 amino acids positioned between the catalytic site and the rim (Tables S1 and S2 in Supplementary Materials) [33].

**Figure 10:**
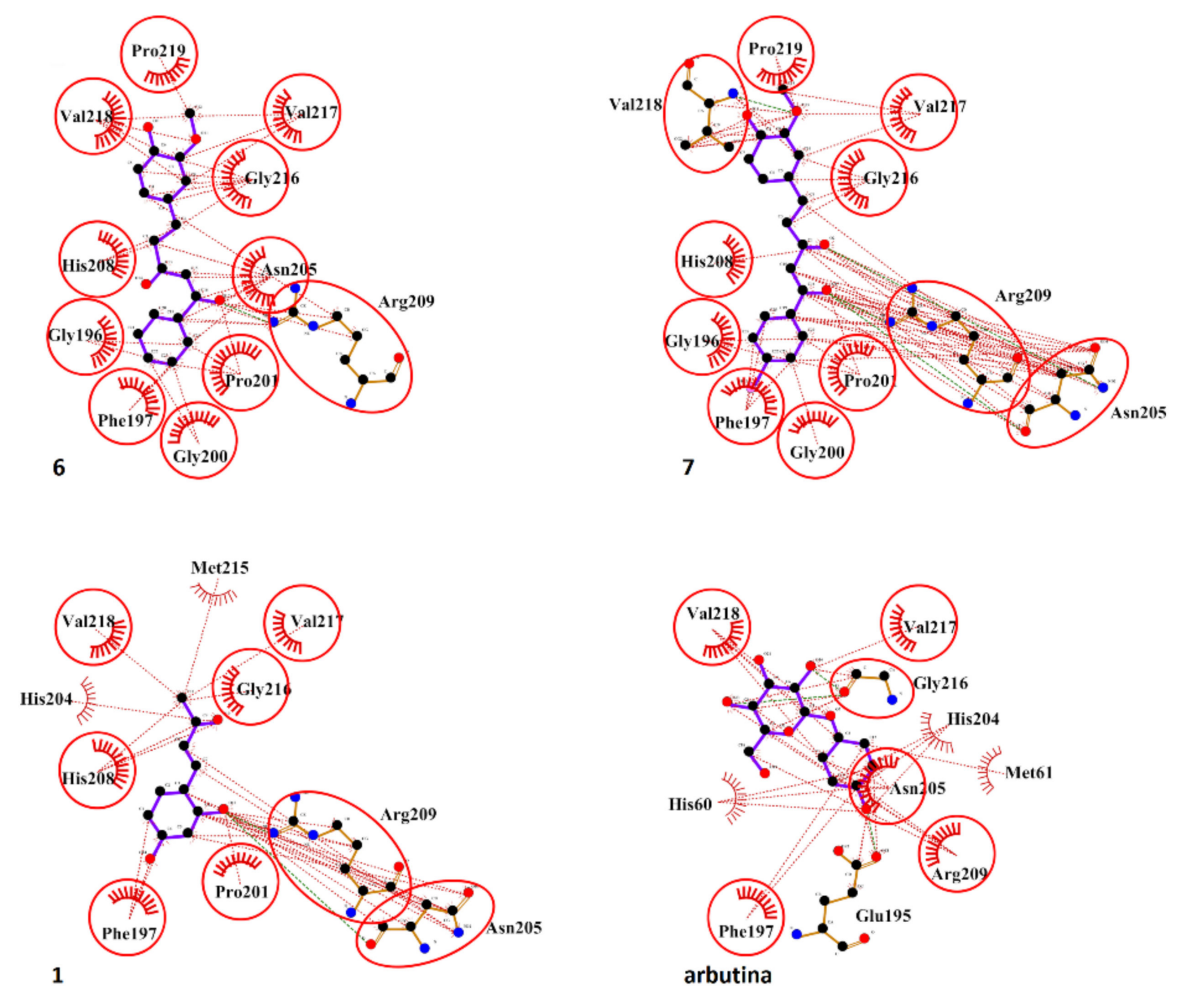
Hydrophobic interactions of the lowest docking poses of compounds **6**, **7**, **1** and arbutin with the catalytic site of *B. megaterium* tyrosinase (3NM8) performed with LigPlot+ [34].

With binding constants ranging μM values, the most populated pose of arbutin and compounds **1**, **6** and **7** interact, to a different extent, with a set of hydrophobic amino acid residues Phe197, Asn205, Arg209, Gly216, Val217, Val218 and, except arbutin, with His208 (Figure 10).

All amino acids are positioned at the entrance of the channel facing the catalytic site, in particular amino acids Glu195, Phe197, Asn205, His208, Arg209 and Val218. In comparison with arbutin and compounds **1** and **6**, strong hydrophobic interactions were evidenced for compound **7**. Compound **7** differs from compound **6** through the presence of one bromine atom in *para*-position to the second phenyl ring. The presence of the halide provides a different conformation of the molecule, which is oriented with the bromine towards Phe197 (stacking interactions) with the keto-enol group in *syn*-conformation. This conformation favors stronger interactions with Phe197, Asn205, Arg209 and Val218 in comparison with that exerted with compound **6** (Figure 10). Compounds **1**, **6**, **7** and arbutin activate H-bonds with Asn205 and Arg209.

**Figure 11:**
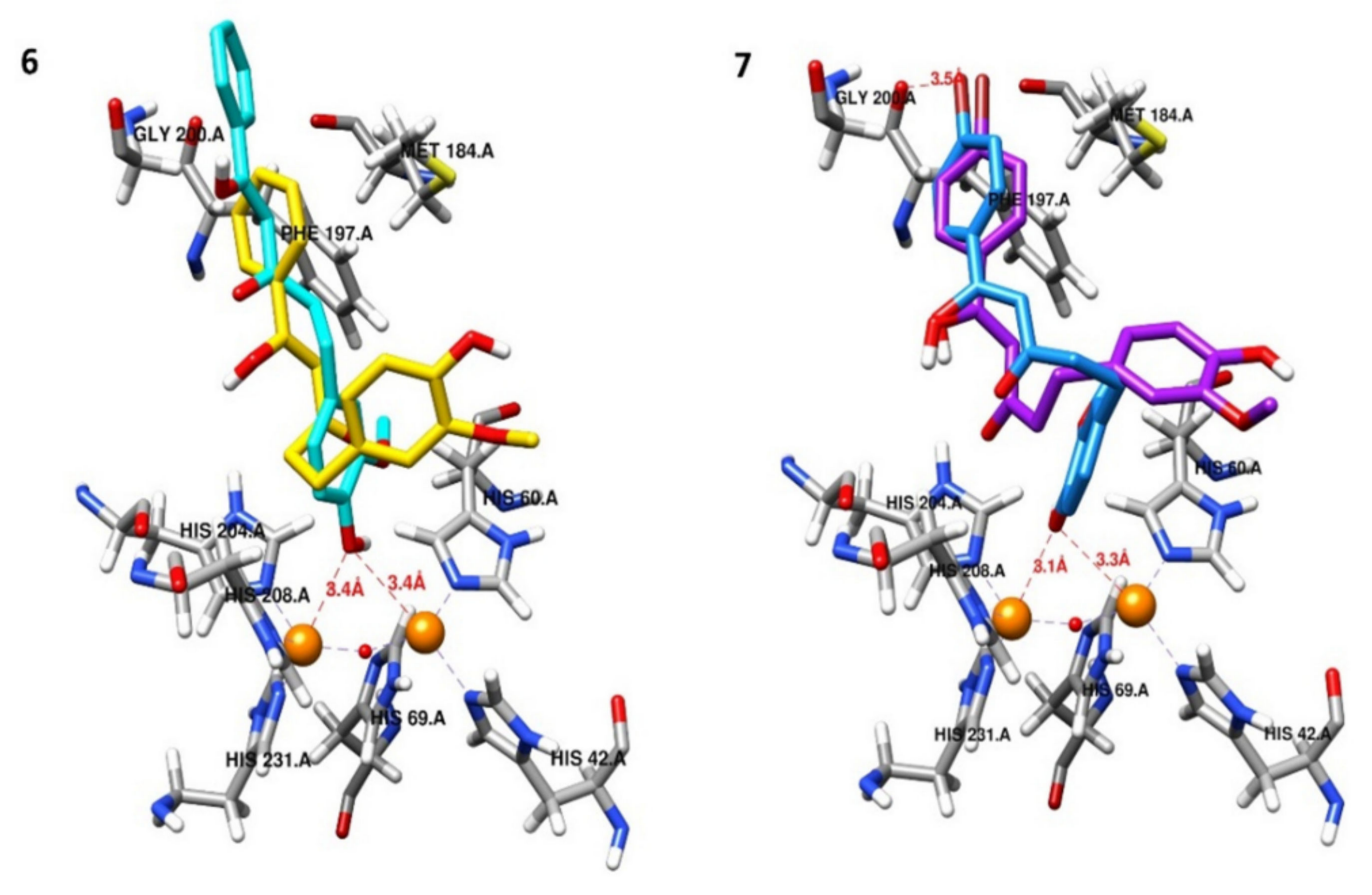
Representation of the two best docking poses of compound **6** and **7** facing the catalytic site where histidines and copper ions are represented at the bottom with sticks and balls, respectively. On the **left**: lowest energy (cyan) and the most populated pose (yellow) of compound **6**. On the **right**: lowest energy (light blue) and the most populated pose (purple) of compound **7**.

Concerning compounds **6** and **7**, the best conformations of the molecule are always oriented with the guaiacyl ring that points to the catalytic site at a distance from the copper ions in the range from 3.1 to 3.4 Å for both compounds. In the conformation at the lowest energy, both compounds **6** and **7** form a binding complex activated by strong interactions with the oxygen of the phenol-OH unit and the two copper ions (Figure 11).

**Figure 12:**
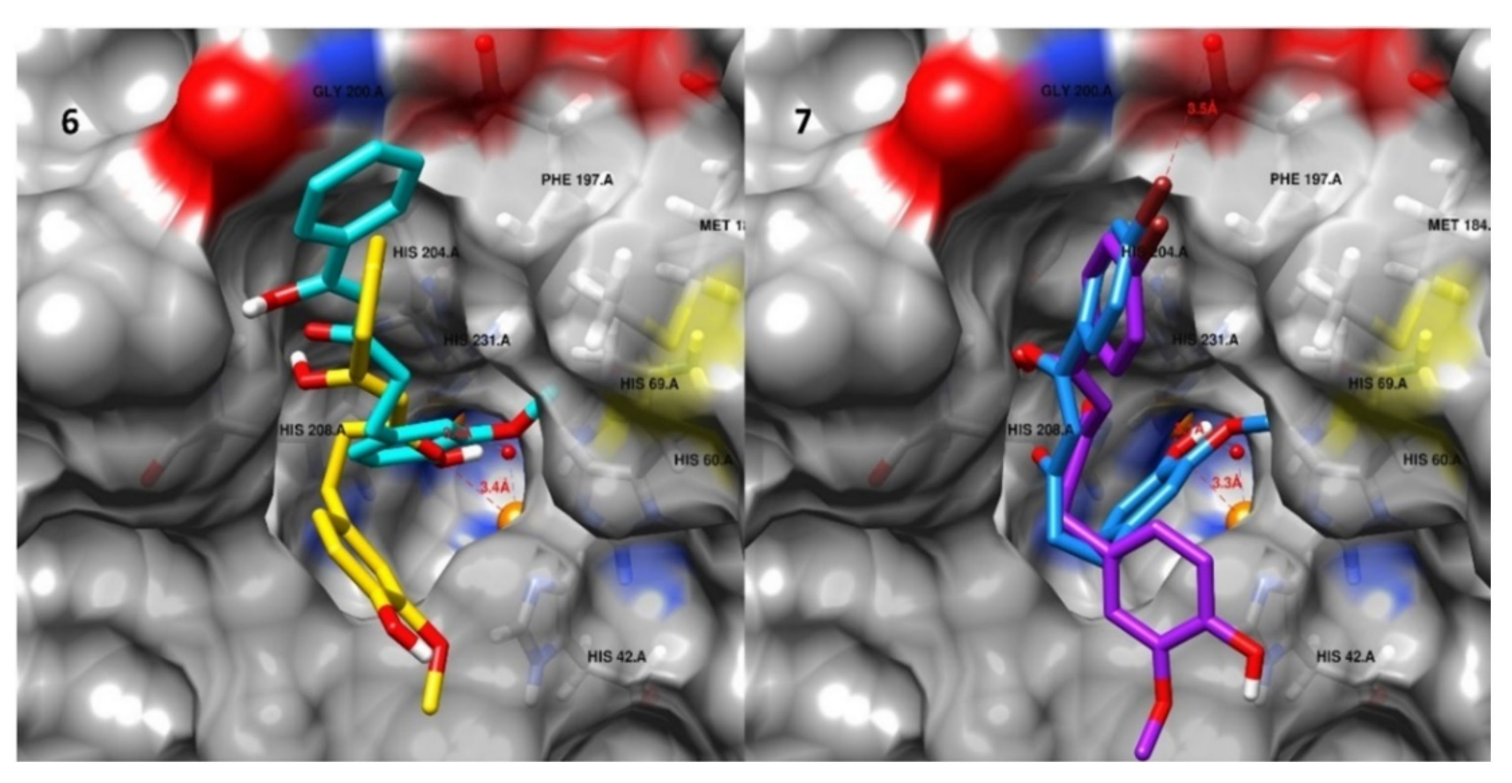
Representation of the best docking poses of compounds **6** and **7** surrounding the catalytic site where one copper ion is represented as orange ball. On the **left**: lowest energy (cyan) and the most populated pose (yellow) of compound **6**. On the **right**: lowest energy (light blue) and the most populated pose (purple) of compound **7**.

Two strong interactions were measured in the two best docking poses of **7** with Phe197: one involved halogen bonding (σ-hole) between the bromo-substituent and the oxygen of the amino acid and the other dealt with a π-π interaction between the brominated phenyl ring of compound **7** and the phenyl ring of Phe197. No similar interactions were evidenced for compounds **1**, **6** and arbutin, even though a weaker stacking binding was evaluated for this compound (Figure 12, Figures S8 and S9 in Supplementary Materials).

## 3. Discussion

We report here the synthesis of two tyrosinase inhibitors featured by 1,3-diketo function and hydroxylated phenyl core to enhance tyrosinase inhibition and to increase cell viability and anti-melanogenic activity for an application both in melanogenesis disorders and skin whiteners. We also prepared compound **1**, a well-known tyrosinase inhibitor [26] that was used as a control. This study follows our recent paper [27], where a series of novel multitarget tyrosinase and laccase enzyme inhibitors, featured by a phenylpropanoid and hydroxylated biphenyl core, were prepared under straightforward synthetic procedures. The chemical structure of compounds **6** and **7** featuring a guaiacyl group and an aliphatic chain in *para*-position to the phenyl OH group contains a keto-enol form ending with a phenyl unit. We modified the geometrical feature of the aliphatic chain by introducing a 1,3-diketo function (keto-enol form), as in the curcumin structure, because this function is susceptible to electron transfer reactions with the amino acid residues of the tyrosinase catalytic site. In the design of compounds **6** and **7**, we focused on functional groups with wide delocalization of π-electrons that activate, as Michael acceptors, electron transfer reactions with nucleophilic species present in amino acids residues of tyrosinase. Moreover, we selected a guaiacyl unit that is generally acknowledged as a safe pharmacophore in the design of tyrosinase inhibitors [15,16,21]. Compound **7** bears a bromine atom in the second phenyl ring. By virtue of the lipophilicity of halides, bromo-substituents are generally used in the design of biologically active compounds, since the presence of halogen enhances membrane permeability and metabolic stability along with acceptable viability [35,36]. From a chemical point of view, halogen bonding acts as a donor in a non-covalent interactions (σ-hole) with a Lewis base (acceptor) with a strength comparable to that of a weak or moderate hydrogen bond [37,38]. Moreover, a bulky electron-withdrawing group in *para*-position to the phenyl ring would increase the inhibitory activity [15]. Molecular docking of compound **7** with tyrosinase protein evidenced these behaviors in comparison to compound **6** lacking in bromo-substitution. Stronger hydrophobic interactions of **7** with Asn205, Arg209 and Phe197 in comparison with those exerted with compounds **1**, **6** and arbutin were evidenced. This set of amino acids is crucial in the stabilization and orientation of the substrate. In fact, it is suggested that the entrance of the substrate into the catalytic site involves the expansion or contraction of the channel volume in virtue of the interactions that the substrate activates with the adjacent amino acid residues [18,39]. The high conformational flexibility of Arg209 provides stabilization of the different substrate conformations, therefore the interactions of **7** with Arg209 would disturb the entrance of the ligand. This is in accordance with the amperometric data, which evidenced for compound **7** a mixed mode of competition in the diphenolase activity (L-DOPA). Compound **7** would bind the catalytic center and a peripherical site likely identified in the rim of the catalytic channel where Asn205, Arg209 and Phe197 play a key role in the enzymatic mechanism, similarly to the binding mode of kojic acid (KA) in *B. megaterium* tyrosinase [PDB file: 3NQ1 and 3NM8] [17].

Recently, it has been suggested that both monophenols and *o*-diphenols bind firstly to the same copper ion since a reorganization of the phenolate around the two copper ions occurs in the diphenolase form [19]. Both copper ions interact with an internal molecule of water involved in the biochemical pathway. Asn205 plays a crucial role in the monophenolase activity because it stabilizes the binding of the substrate through the molecule of water positioned in the catalytic site [19]. The presence of an inhibitor, such as **7**, would hinder the stabilization of the substrate promoted by the molecule of water.

The strong hydrophobic interactions that compound **7** and, to a lesser extent arbutin, activate with Val218 suggest the role that the amino acid plays in the melanogenic process, since compound **7** works as a strong pigmentation inhibitor as arbutin does.

Val218, in virtue of its small size and conformational flexibility, controls the entrance of the ligand into the active site, allowing the monophenolase reaction to occur [18]. This amino acid, through hydrophobic interactions with His208, orients the substrate towards the copper ion involved in the attack. Compound **7** would hamper the interactions between substrate and Val218, compromising the main function of the amino acid in directing the substrate correctly and therefore altering the enzymatic process in the following reaction steps.

The guaiacyl unit of compounds **6** and **7** might contribute to the inhibitory activity of the molecules, disturbing the entrance and stabilization of the substrate. The best poses of both compounds point to the catalytic site activating stronger hydrophobic interactions and Cu-binding complexes than that of the hydroxylated phenyl units of compound **1** and arbutin.

Since conventional inhibitors suffer from toxicity and a lack of efficacy, all compounds were, in advance, subjected to cytotoxicity assays. Compounds **6** and **7** did not show significant toxicity on human fibroblasts, nor melanoma cell lines at a concentration lower than 100 µM. This behavior is comparable to that of arbutin. Interestingly, compound **7**, unlike **6**, did not affect normal fibroblasts growth, even at 100 µM, indicating that it might not be toxic for normal human cells, while it reduces the viability of B16F10 and LCP melanoma cell lines at 100 µM to a greater degree than compound **6**. Moreover, our results showed that neither the proliferation of melanoma cell lines nor that of fibroblasts were inhibited by the treatment with 1–10–100 µM of compound **1**.

Toxicity and protection of compounds **1**, **6** and **7** were also evaluated on PC12 cell cultures: molecules **6** and **7** were found to be non-toxic if compared to the control, with cell viabilities around 80%, while compound **1** showed to decrease cell viability compared to the control, with a slight toxicity towards cell cultures starting from 10 μM onwards.

Once the low toxicity of the compounds on cell lines has been determined, the capability of molecules to inhibit the activity of tyrosinase was evaluated using a UV-Vis spectrophotometric method and by biosensors.

Compound **1** showed to be a good tyrosinase inhibitor, strongly decreasing the enzyme activity, especially in the presence of L-Tyr as a substrate. Moreover, spectrophotometric results showed that compounds **6** and **7** were weak tyrosinase inhibitors, especially in the presence of dopamine as a substrate, and that the presence of a bromine atom seems to have no influence on the enzymatic inhibition. In the presence of dopamine at 2 μM concentration, compound **7** was slightly more effective than **6**.

As previously demonstrated [27], amperometric enzyme-based biosensors were used to highlight the mechanism of enzyme inhibition exerted by the studied compounds. Although the definition of the interaction is complex due to the enzyme immobilization, in these experiments, the biosensors also proved to be effective in identifying the type of inhibition determined by the presence of the investigated compounds.

Our previous work [27] and the literature data [26] have already demonstrated, using different techniques, that compound **1** is a competitive inhibitor of the diphenolase activity. In this work, amperometric biosensors have highlighted the same type of inhibition for compound **1**, demonstrating the efficacy of such devices for the study of inhibition mechanisms. By means of the same amperometric devices, compound **6** has shown to be a competitive inhibitor in the diphenolase activity, while compound **7** a mixed inhibitor. Compounds **1** and **6** act by mimicking the enzyme substrate and inhibit the substrate binding with the catalytic site. We are not able to provide an exhaustive explanation on the different inhibition mode of compound **7** in comparison with **1** and **6**. We can only speculate that the mixed inhibition mode of **7**, evidenced by electrochemical detection, would be due to the possibility of the compound to bind the free enzyme and the enzyme-substrate complex with the same equilibrium constant. In silico, this inhibition mode might be evidenced with a strong interaction with Phe197, Asn205, Arg209 and Val218, especially with Phe197, because no similar interactions were evidenced for compounds **1** and **6**. We did not exclude an allosteric site for compound **7** located external to the selected docking grid that circumscribes the interaction area of the catalytic site. It has also been postulated that these compounds are able to trap the *o*-dopaquinone intermediate in virtue of the nucleophilic feature of the phenolic OH group. Like most polyphenolic compounds [40], compound **7** also exerted mixed inhibition on the tyrosinase enzyme by binding not only to a free enzyme but also to the enzyme-substrate complex. All the studied molecules showed very low IC_50_ values, comprising between 11 and 24 nM, by means of electrochemical experiments. The IC_50_ values represent the inhibitor concentration by which the tyrosinase activity was reduced by 50% according to dose-response curves. Regarding compound **1**, these data were perfectly in line with those observed previously [27].

The difference in the ability to exert a tyrosinase inhibition observed in the two analytical assays for compounds **1**, **6** and **7**, (UV-Vis spectroscopic method and amperometric enzyme-based method) could be explained in terms of the different experimental environment that could severely influence tyrosinase activity. In fact, in the biosensor probe the enzyme is adsorbed on two lipophilic layers, while in the spectrophotometric assay it forms an aqueous solution.

Moreover, all the compounds were tested against oxidative damage induced by H_2_O_2_, with compound **6** resulting the most interesting, as it was protected from oxidative insult at all tested concentrations. Compounds **1** and **7** resulted less effective protective agents; in fact, they were able to restore H_2_O_2_ only at concentrations of 1µM and 10µM, respectively.

Melanin content assays were performed to determine the anti-melanogenic properties of compounds **1**, **6** and **7**. The presence of the bromine atom in compound **7** appears to be a determining factor of anti-melanogenic activity. Indeed, while compound **7** induced a significant decrease in melanin content in all human melanoma cells, compound **6** did not show any activity on LCP human melanoma cells (Figure 7). The inhibitory effect of compound **7** on melanin production was significant and comparable to that of arbutin on human melanoma cells, even if the concentration of **7** was much lower (20 µM vs. 250 µM of arbutin).

These data are in accordance with the greater antioxidant activity detected by amperometric experiments of compound **7** compared with **6**. The higher melanin synthesis inhibition of **7** compared to compound **6** could be due to the bromine atom in *para* position of the second phenyl ring, which allows a more efficient delocalization of the radical species [41]. It is in fact known that the introduction of halogen atoms enhances the cellular uptake of molecules [35]. Halogen bonds are in fact noncovalent interactions, where halogen atoms act as electrophilic species interacting with Lewis bases. These interactions are relevant in biochemical systems, being increasingly explored in drug discovery, mainly to modulate protein–ligand interactions. It is also known that modifying a molecule with a bulky electron-withdrawing group in para-position on an aromatic ring would be essential to the tyrosinase inhibitory activity [15]. Indeed, it is known that the melanin production process generates hydrogen peroxide (H_2_O_2_) which induces significant oxidative stress in melanocytes. Moreover, scavengers and ROS inhibitors can downregulate UV-induced melanogenesis [42].

These results and the low toxicity of compound **7** on human fibroblasts and on melanoma cells suggest that this compound could be promising for therapeutic or cosmetic applications.

Our studies include only three structures, undoubtedly worthwhile in the future to extend the number of compounds with other halides in para-position that act as a donor in a non-covalent interaction (σ-hole) with a Lewis base (acceptor) with a strength comparable to that of a weak or moderate hydrogen bond. Moreover, no-halide substituents with an electron-withdrawing group in para-position with different steric hindrance should be evaluated. Considering the interesting tyrosinase inhibition activity and low toxicity in fibroblast cells of compounds **6** and **7**, anti-melanogenic activity would be assessed in zebrafish before it is tested on human skin.

## 4. Materials and Methods

### 4.1. General Methods and Reagents

Unless otherwise stated, starting materials and reagents were obtained from Sigma Aldrich, Munich, Germany and were used without further purification. Melting points were determined on a Büchi 530 apparatus and are uncorrected. All ^1^H-NMR and ^13^C-NMR spectra were recorded in CDCl_3_ (if not otherwise indicated) solution at 399.94 MHz and 75.42 MHz, respectively, with a Varian VXR 5000 spectrometer Varian, (Palo Alto, CA, USA). Chemical shifts are given in ppm (δ); multiplicities are indicated by s (singlet), d (doublet), t (triplet), q (quartet), m (multiplet) or dd (doublet of doublets). Elemental analyses were performed using an elemental analyzer model 240 C (Perkin-Elmer, Walthan, MA, USA). Acetone was freshly distilled from CaCl_2_. Flash chromatography was carried out with silica gel 60 (230–400 mesh) (VWR, Radnor, AF, USA) eluting with an appropriate solution in the stated *v*:*v* proportions. All reactions were monitored by analytical thin-layer chromatography (TLC) with 0.25 mm thick silica gel plates (60 F 254) (Sigma Aldrich, Munich, Germany). Melting points were determined on a 530 apparatus (Büchi, Flawil, Switzerland) and are uncorrected. The purity of all new compounds was judged to be >98% by ^1^H-NMR spectral determination.

#### 4.1.1. Synthesis of Inhibitors

*(E)-4-(2,4-dihydroxyphenyl)but-3-en-2-one***1**: to a stirred solution of 2,4-dihydroxybenzaldehyde (1.5 g, 10.8 mmol) in acetone (20 mL), an aqueous solution 1N of NaOH (40 mL, 40 mmol) was added dropwise. The mixture was stirred under microwave irradiation at 80 °C for 2 h. After rotoevaporation of the solvent, water and 10% hydrochloric acid were carefully added. The solution was extracted with ethyl acetate, dried over anhydrous sodium sulphate and evaporated. The crude was purified by flash chromatography using a 1:1 mixture of petroleum ether:ethyl acetate as eluent to give compound **1** as brown solid (1.17 g, 61%): mp 117–119 °C (lit. [26] 118–120 °C); ^1^H-NMR (CD_3_COCD_3_) δ 2.23 (s, 3H), 6.42 (dd, *J* = 2.0, 8.4 Hz, Ar, 1H), 6.46 (d, *J* = 2.0 Hz, Ar, 1H), 6.68 (d, *J* = 16.4 Hz, 1H), 7.49 (d, *J* = 8.4 Hz, Ar, 1H), 7.81 (d, *J* = 16.4 Hz, Ar, 1H); ^13^C-NMR (CD_3_COCD_3_) δ 26.33, 102.70, 108.15, 113.71, 123.87, 129.95, 138.41, 158.31, 160.93, 197.05. Anal. Calcd. for C_10_H_12_O_3_: C, 66.65; H, 6.71 Found: C, 66.70; H, 6.68.

#### 4.1.2. General Procedure for the Synthesis of Compounds **6** and **7**

To a solution of zingerone (5.1 mmol, 1 eq.) in dry tetrahydrofuran (20 mL), solid anhydrous sodium ethoxide (15.3 mmol, 3 eq.) was added under N_2_. The reaction mixture was stirred at room temperature for 30 min. The appropriate ester (ethyl benzoate for **6** or ethyl 4-bromobenzoate for **7**) (7.6 mmol, 1.5 eq.) was added and the solution was stirred at room temperature for 12 h and neutralized with 10% aqueous hydrochloric acid. The organic phase was extracted twice with diethyl ether (2x30 mL), dried over anhydrous sodium sulphate and evaporated to obtain **6** or **7,** respectively, as brown solids that were purified by flash chromatography, using dichloromethane as eluent.

*(Z)-1-hydroxy-5-(4-hydroxy-3-methoxyphenyl)-1-phenylpent-1-en-3-one* **6**: light yellow solid, 88%; mp 77–78 °C (lit. [29], 76–78 °C); ^1^H-NMR (CDCl_3_) δ 2.71 (t, *J* = 6.8 Hz, 2H), 2.95 (t, *J* = 6.8 Hz, 2H), 3.86 (s, 3H), 5.42 (bs, 1H), 6.15 (s, 1H), 6.71 (dd, *J* = 2.0, 8.4 Hz, Ar, 1H), 6,74 (d, *J* = 2.0 Hz, Ar, 1H), 6.84 (d, *J* = 8.4 Hz, Ar, 1H), 7.44 (m, Ar, 2H), 7.51 (m, Ar, 1H), 7.86 (m, Ar, 2H); ^13^C-NMR (CDCl_3_) δ 31.41, 41.36, 55.86, 96.44, 111.01, 114.37, 120.85, 126.00, 128.63, 132.33, 132.63, 134.9, 144.03, 146.44, 183.25, 195.89; Anal. Calcd for C_18_H_18_O_4_: C, 72.47; H, 6.08; Found: C, 72.52; H, 6.03.

*(Z)-1-(4-(bromo)phenyl)-1-hydroxy-5-(4-hydroxy-3-methoxyphenyl)pent-1-en-3-one***7**: light yellow solid, 63%; mp 98–99 °C; ^1^H-NMR (CDCl_3_) δ 2.70 (t, *J* = 6.8 Hz, 2H), 2.93 (t, *J* = 6.8 Hz, 2H), 3.86 (s, 3H), 5.47 (s, 1H), 6.01 (s, 1H), 6.71 (dd, *J* = 2.0, 8.8 Hz, Ar, 1H), 6,73 (d, *J* = 2.0 Hz, Ar, 1H), 6.83 (d, *J* = 8.8 Hz, Ar, 1H), 7.57 (m, Ar, 2H), 7.71 (m, Ar, 2H); ^13^C-NMR (CDCl_3_) δ 31.36, 31.33, 55.87, 96.40, 111.09, 114.43, 120.85, 127.14, 128.49, 131.91, 132.50, 133.73, 144.07, 146.48, 182.08, 196.11; Anal. Calcd for C_18_H_17_BrO_4_: C, 57.31; H, 4.54; Found: C, 57.26; H, 4.49.

### 4.2. Measurement of Tyrosinase Inhibitory Activity

#### 4.2.1. Mushroom Tyrosinase Spectrophotometric Assay

The capability of molecules to inhibit the oxidation of either Tyr or dopamine by the tyrosinase/O_2_ oxidizing system was monitored by a UV-Vis spectroscopy method, carried out in a Shimadzu UV-Visible UV-2501 spectrophotometer (Tokyo, Japan) using a Helma (Mülheim, Germany) duple chambers UV-cell (2 × 4.375 mm). Briefly, 880 µL of a substrate solution (L-Tyr 2 mM, dopamine 17.6 µM) and 900 µL of a mushroom tyrosinase solution (2000 U/mL, Sigma-Aldrich) both dissolved in 50 mM phosphate buffer (pH 6.8) were separately introduced in the separated duple UV-cell chambers. Then, 20 µL of an appropriate DMSO stock solution of inhibitors (0.18 or 0.018 mM, respectively) were added to the chamber containing the substrate solution. The tyrosinase activity in the absence of inhibitors was detected, adding 20 µL of DMSO. UV-Vis spectra were recorded in the 250–800 nm wavelength range with a 2.0 nm slit and fast speed before starting the reaction. Thereafter, the solutions were mixed and UV-Vis spectra were acquired at different time points after mixing. Data processing was performed with Shimadzu UV Probe and Origin Pro 2019 (v. 9.60) softwares Origin Lav Corporation, (Northampton, MA, USA).

Purchased mushroom tyrosinase was used without further purification. L-tyrosine, dopamine and tyrosinase solutions were filtered through a 0.45 μm syringe filter, aliquoted in Eppendorf tubes, and stored in freezer. The concentration of substrates and of enzyme solution was determined spectrophotometrically (Ɛ_Tyr_ at 274.6 nm = 1420 M^−1^·cm^−1^; Ɛ_dopamine_ at 280 nm= 2680 M^−1^·cm^−1^; Ɛ_tyrosinase_ at 280 nm = 1426 M^−1^·cm^−1^).

Tyrosinase-dependent substrate oxidation was assessed evaluating the absorbance values before the mixing and after 24 min

%inhibition = 1 − [(D − C)/(B − A)] * 100

where C and D are the absorbance values at 475 nm in the presence of an inhibitor before the mixing and after 24 min, respectively, while A and B are the corresponding values in the absence of an inhibitor [27,30].

#### 4.2.2. Preparation of Tyrosinase Biosensors

All the chemical compounds used for biosensors’ preparation and characterization were purchased by Sigma-Aldrich, Milano, Italy. Tyrosinase-based biosensors (Figure S10 in Supplementary Materials) were constructed by starting from a Teflon™-insulated silver wire (30 mm in length; Ø = 125 μm; Advent Research Materials, Eynsham, England), as previously described [27,43]. Shortly, 1 mm of the silver wire was uncovered and then put into a portion of a silica capillary (10 mm in length; I.D. Ø = 180 μm, Polymicro Technologies, Phoenix, AZ, USA) partially filled with graphite-loaded (55% *w*/*w*) epoxy resin, obtained by mixing 850 mg of graphite and 500 mg of Araldite-M and with the addition of 200 mg of hardener. An initial disc electrode of carbon-composite was realized (Ø = 180 μm; area: 2.5 × 10^−4^ cm^2^) by replenishing the silica capillary tubing with the mixture. The silver wire was needed for guaranteeing a good electrical contact. Sensors were then left for 24 h at 40 °C and after, the surface was cleaned using a high-speed drill (Dremel^®^ 300) with an aluminum oxide grinding wheel. On top of the electrode surface a 1 µL of Tyrosinase solution (10 U/µL) was loaded. After the evaporation of water at room temperature, the biosensors were rapidly dipped in a polyurethane solution (1%) in order to ensure the enzyme on the electrode surface and left for 30 min at room temperature. Then, the solvent biosensors were put in PBS pH = 6.0 overnight to allow for current stabilization.

#### 4.2.3. Biosensor Calibration Protocol

The electrochemical experiments were performed at room temperature in a classical three-electrode electrochemical cell consisting of four tyrosinase biosensors as working electrodes, a reference electrode Ag/AgCl in NaCl, 3.0 M, BASi (West Lafayette, IN, USA) and a large surface steel wire as the auxiliary electrode, as before described [44].

In order to verify tyrosinase biosensors’ performances, preliminary cyclic voltammetry experiments were conducted in PBS at pH = 6.0, using dopamine as a reference compound, so as to define the working reduction potential of the quinone, obtained by the oxidation of the amine. Then, cyclic voltammetries of selected inhibitor compounds were performed in the same conditions as dopamine to evaluate any possible peak overlapping in the reduction region and to assess any possible interfering current. Voltammograms (data not shown) were obtained in a range of potentials, comprising between −0.5 V and +0.5 V at a scan rate of 100 mV s^−1^.

For in-vitro calibrations and for inhibition evaluation experiments, constant potential amperometry (CPA) was used by means of a fixed potential of −50 mV against an Ag/AgCl reference electrode, through a four-channel potentiostat eDAQ Quadstat, e-Corder 410, eDAQ Europe, (Warsaw, Poland) and the software Chart v. 5.5, eDAQ Europe, (Warsaw, Poland). Once the stabilization of the baseline current was reached, increasing volumes of a stock solution of dopamine (1 M) were injected in the cell in order to obtain different concentrations between 0 and 140 mM. Biosensors were then characterized in their kinetic parameters (V_MAX_ and apparent K_M_) by means of Michaelis–Menten kinetics, in order to determine the possible inhibitory effects of the studied compounds. Statistical differences in parameters were assessed by means of *t*-test (*p* < 0.05). The GraphPad Prism software, v. 9.03, was used for the calculation of enzymatic kinetic parameters and IC_50_ values, as well as to perform the *t*-test.

#### 4.2.4. Biosensor Inhibition Protocols

For each inhibitor, a group of tyrosinase biosensors (n = 4) was built. The inhibition of selected compounds was assessed by means of two different protocols. The first were used in order to evaluate the IC_50_ values and the lowest inhibitory concentration of each evaluated compound, as previously defined [27]. In brief, a fixed concentration of dopamine (50 µM) was injected in the electrochemical cell containing 10 mL of PBS pH = 6.0, and the reduction current of the obtained quinone was monitored at a fixed potential of −50 mV vs. Ag/AgCl. After having obtained a stable baseline, defined volumes of a stock solution of each inhibitor (10 mM) were added in the cell up to the desired concentration.

#### 4.2.5. Kinetic Analyses of the Compounds

To assess the inhibition types, the second protocol was used. A preliminary calibration with dopamine, in a range comprising between 0 and 150 mM, was made in order to determine the values of V_MAX_ and K_M_. The same calibration protocol was performed after exposing the biosensors for 30 mins to twice the lowest inhibiting concentration of each inhibitor, in order to guarantee the inhibition of enzymatic activity. So, biosensors were exposed to the inhibitor for 30 min and after having obtained a stabile baseline, the same calibration with dopamine was performed as before and the value of V_MAX_ and K_M_ were then calculated. Variations in both kinetic parameters were then analyzed by using the Lineweaver–Burk plot in order to categorize the type of inhibition induced by the tested molecule. The statistical difference in V_MAX_ and K_M_ was calculated by means of a paired *t*-test (*p* < 0.05) prior to the biosensor exposure to the inhibitor and then thereafter.

### 4.3. Antioxidant Activity of the Compounds via Amperometric Assay

All the chemical compounds used for microsensors preparation and characterization were purchased by Sigma-Aldrich, Milano, Italy. In order to evaluate the possible antioxidant activity of the investigated compounds, platinum-based electrodes (Figure S11 in Supplementary Materials) were realized by cutting a 3 cm portion of Teflon^®^ insulated Pt/Ir wire (125 µm Ø, Advent Research Materials, Suffolk, U.K.) from which an active cylinder of 1 mm was obtained by removing the Teflon^®^ insulating layer from one end. The active surface was then appropriately modified by electrodepositing a poly-ortho-phenylene polymer (PPD) nanometric layer, as previously reported [44,45]. Hydrogen peroxide was used as the oxidant compound to verify the antioxidant activity of the selected inhibitors. Previously, it was demonstrated that the H_2_O_2_ current monitored at the platinum electrode surface could be depleted by a concurrent homogeneous redox reaction due to the presence of reducing compounds [31]. Consequently, the PPD polymer was layered to avoid the electrochemical monitoring of any electroactive compounds excepting H_2_O_2_. The shielding capabilities of the polymer against inhibitors were previously verified by adding increasing concentrations of both compounds in the electrochemical cell, after the electro polymerization (data not shown). The applied potential was the same as that used for the anodic oxidation of H_2_O_2_ (+700 mV vs. Ag/AgCl).

Antioxidant activity of selected inhibitors was evaluated against H_2_O_2_. The protocol was the following: after the electropolymerization of PPD, obtained from a stock solution of 300 mM of OPD (o-phenylenediamine dihydrochloride) [46], a fixed concentration of H_2_O_2_ (200 µM) was added in the electrochemical cell. After having reached the current steady state, increasing concentrations of inhibitors were added, ranging from 0 to 200 µM, dependently on the solubility of the compounds’ stock solution in PBS. The decrease in H_2_O_2_ current was monitored and evaluated.

### 4.4. Cell Culture

#### 4.4.1. Cell Lines

The rat pheochromocytoma cell line PC12 (ATCC^®^ CRL-1721™) [47] together with the two human malignant melanoma cell lines LCP-mel (RRID:CVCL_7053) and PNP-mel (RRID:CVCL_G320) [48] have been used as experimental models for our study. The last are primary human melanoma cells derived from tumor biopsies and were kindly donated from the Istituto Dermopatico dell’Immacolata (IDI, Rome, Italy). As a non-tumor control, we used a human fibroblast cell line from a healthy donor, the BJ cell line (CRL-2522™- ATCC^®^, Manassas, VA, USA). The mouse melanoma cell line B16F10 (ATCC^®^ CRL-6223™ ATCC^®^, Manassas, VA, USA) was also used, only for the melanin content assays.

Human malignant melanoma cell lines were cultured in RPMI medium with stable glutamine, supplemented with 10% fetal bovine serum (FBS) and penicillin/streptomycin (1 U/mL) (all from Euroclone^®^, Pero, Italy), (complete medium), in a humidified atmosphere with 5% CO_2_, at 37 °C.

PC12 cells (passage 12–25) were maintained in atmosphere of 5% CO_2_/95% humidified air at a fixed temperature of 37 °C and cultured in 60 mm Ø plastic plates with Dulbecco’s modified Eagle’s medium (DMEM/F12) added with 10% Horse Serum (HS), 5% Fetal Bovine Serum (FBS) and 1% of penicillin/streptomycin.

#### 4.4.2. Toxicity of Compounds and Their Protective Activity on PC12 Cells

According to protocols present in the literature [27,47], PC12 cells were treated for 24 h with different concentrations of the selected molecules ranging from 1 to 40 μM in order to evaluate their toxicity. Thereafter, to estimate the possible compounds’ protection properties, cells were treated with hydrogen peroxide (100 μM) in association with increasing concentration of inhibitors, ranging from 1 up to 40 μM.

At the conclusion of the exposure time of each compound, the cell viability was evaluated by means of MTT (3-(4,5-dimethyl-thiazol-2-yl)-2,5-diphenyltetrazoliumbromide) assay. In this study, 1 mg/mL of MTT was added to each sample and incubated for 4 h at 37 °C. As known, only viable cells are able to convert the soluble dye MTT into the insoluble formazan crystals. After the incubation period, the MTT supernatant solution was removed. Then, the cells were washed in phosphate-buffered saline (PBS) and centrifuged, and the pellet was then dissolved in 2 mL of isopropanol. The solution was centrifuged at 4000 rpm for 5 min and the absorbance for each sample was evaluated by means of a Bauty Diagnostic Microplate Reader at 578 nm. All experiments were performed in 24 well plates (1 × 10^5^ cells/mL/well) and repeated in triplicate.

#### 4.4.3. Toxicity of Compounds: Cell Proliferation Assays on Melanoma Cells

Cells were seeded in 96 well plates, in RPMI culture medium with stable glutamine, supplemented with 10% FBS and penicillin/streptomycin (1 U/mL), and grown in a humidified atmosphere with 5% CO_2_, at 37 °C. After 24 h, the medium was replaced by fresh complete medium supplemented or not (control) with increasing concentrations (1–10–100 mM) of either **1**, or **6**, or **7** compounds. Cells were grown up to 48 h. Cell viability was determined by MTT assay as described previously [49,50]. All experiments were performed in triplicate and repeated at least three times.

#### 4.4.4. Inhibition of Melanin Synthesis: Melanin Quantification Assay

We followed a previously described method with minor modifications [51]. Briefly, cell lines were seeded in triplicate at a density of 2 × 10^5^ cells/well in a 24-well plates culture with 500 μL of complete medium/well (all components from Euroclone, Pero, Italy). After 24 h, the medium was replaced by fresh medium containing αMSH (alfa Melanocyte Stimulating Hormone, 10 nM) (#M4135, Sigma Aldrich, St. Louis, MO, USA), and Arbutin (250 μM) (#A4256, Sigma Aldrich, St. Louis, MO, USA) or either compounds **1**, **6** (100 μM) or **7** (20 μM and 100 μM). Incubation was carried out at 37 °C under 5% CO_2_ atmosphere for 48 h. Following incubation, cells were washed twice with PBS (500 μL each) and then cell pellets were dissolved in 350 μL of 1.0 N NaOH containing 10% DMSO (Sigma Aldrich, St. Louis, MO, USA) and heated at 90 °C for 1 h. NaOH solution were transferred in 96 wells plates and the amount of melanin were spectrophotometrically measured at 405 nm with a microplate reader (Sunrise™ Absorbance Reader-TECAN). All experiments were performed in triplicate and repeated at least three times.

### 4.5. Molecular Modelling and Molecular Docking Procedures

#### 4.5.1. Ligand Preparation

Arbutin and synthetic derivatives (**1**, **6**, and **7**) were constructed with standard bond lengths and angles from the fragment database of GaussView 6.1 [52]. Minimization of structures and an extensive conformer search was performed with the GMMX modules of GaussView 6.1, using the MMFF94 force field with energy windows of 3.5 kcal/mol. Representative minimum energy conformations of each inhibitor were optimized using the Density Functional Theory (DFT), quantum chemistry Gaussian 16W program [53] with B3LYP/6–311G basis set. Virtual analysis was conducted with GaussView 6.1. The crystal structure of *B. megaterium* derived tyrosinase was obtained from the RCSB protein databank (PDB file: 3NM8) [17,54,55]. Kojic acid used in this experiment was extracted from the PDB file: 3NQ1 [17], while the hydrogen atoms were added using the tools of Chimera software, version 1.8 [56]. The atomic charges were assigned using the Gasteiger-Marsili [57] method for the ligand and the protein. The Chimera program was also used for the graphic visualization and analysis of the biomolecules. For docking experiments, AutoDock 4.2.6 docking programs [58] was used. Binding of the compounds was analysed using MGLTools 1.5.7rc1.

#### 4.5.2. Computational Docking Procedures

The starting protein tyrosinase was prepared from the 2.00 Å resolution crystal structure deposited by Sendovski et al. [17] (PDB file: 3NM8). As described in our recent work [33], we used the 3NM8 that is devoid of inhibitor, and we tried to reproduce the 3NQ1. The binding site of 3NM8 was determined by comparing the position of the tyrosinase inhibitor (Kojic Acid) as present in the 3NQ1 X-ray [17,18]. Provided the type of protein-containing inhibitor KA (PDB file: 3NQ1)**,** the docking was performed using a grid of 60 × 60 × 60 points, 0.375 Å spacing, and center −10, 20, −5, in order to circumscribe the interaction area of the catalytic site and therefore improving computation time. Then, considering that crystal structures 3NQ1 and 3NM8 are overlapping, the docking of the compounds was performed using crystal protein 3NM8 lacking the ligand.

Additionally, some tests were run considering the whole enzyme to search for other interesting sites. The crystallographic water molecules and Zn/Cl ions not involved in the catalytic process were stripped. Conversely, the two copper ions and the molecule of water present in the catalytic site were conserved.

Hydrogen atoms were added using the ADT module of the MGL Tools 1.5.7rc1 suite program. The ligands were docked using the Lamarckian genetic algorithm (LGA), with the default grid spacing, treating the docking active site as a rigid molecule and the ligands as flexible, i.e., all non-ring torsions were considered active. The LGA of up to 100 runs was employed with the settings of population size of 150 individuals, maximum number of generations and energy evaluations of 27,000 and 25,000,000, respectively. Estimated inhibition constant (K*_i_*_ex_) is modeled by the equation:

K*_i_*_ex_ =exp [(ΔG_ex_ * 1000)/(R * T)]

where ΔG_ex_ is a semiempirical free energy approximation (derived from molecular mechanics and experimental parameters), R (gas constant) = 1.98719 cal·K^−1^·mol^−1^ and T = 298.15 K. Hydrophobic interactions of the best docking pose of all ligands with the catalytic site of B. megaterium tyrosinase protein (3NM8) were performed with LigPlot+ [34]. Computational modelling experiments were carried out on a HP8100 Workstation and an EXXACT Tensor Server TWS-1686525-AMB with GPU capability.

### 4.6. Statistical Analysis

Data were reported as the mean ± standard errors (SEM). In electrochemical experiments V_MAX_ was expressed as nA, while K_M_ as µM. Data from the microsensor were given as a percentage change in the H_2_O_2_ current. All data were statistically evaluated by using Graph-Pad Prism 9.3 software (San Diego, CA, USA).

## 5. Conclusions

In this study, three curcumin-inspired compounds, **1**, **6** and **7**, were prepared and spectrophotometric, electrochemical, in vitro and in silico analyses were carried out. All compounds were prepared in good yields and with straightforward and sustainable synthetic procedures. All compounds caused a decrease in melanin content, especially in human melanoma cells. In particular, compound **7** showed a melanin inhibitory effect comparable to that of arbutin, a well-known skin whitener, on human melanoma cells, but at much lower concentrations (20 µM vs. 250 µM). Particular attention was paid to the determination of the possible toxic activity of the compounds on two human melanoma cell lines (LCP-mel and PNP-mel), a mouse melanoma cell line B16F10 and on a human normal fibroblasts cell line BJ. Our results showed that the tyrosinase inhibitor **1** as well as **6** and **7**, when used at concentrations lower than 100 µM, did not exert significant toxicity on the tested cell lines. The compounds were also screened on PC12 cells. Compounds **6** and **7** were found to be nontoxic towards PC12 cells at all tested concentrations. On the contrary, compound **1** produced a slight toxicity towards cell cultures starting from a concentration of 10 µM. The synthetized compounds were able to inhibit the activity of tyrosinase enzyme in amperometric enzyme-based biosensors and UV-Vis analyses. In the mushroom tyrosinase spectrophotometric assay, compound **1** showed to strongly decrease the tyrosinase activity, especially in the presence of L-Tyr as a substrate, while in tyrosinase biosensor assay, only compound **7** determined a significative variation in V_MAX_, while all compounds produced significative increases in K_M_ values. These results permitted to determine the basic type of enzyme inhibition for each compound. The antioxidant activity of the selected inhibitors was evaluated against H_2_O_2_ in amperometric experiments. Compound **7** was about three times more effective compared to compounds **1** and **6**. Molecular docking of compound **7** showed a higher affinity to tyrosinase protein in comparison to compounds **6** and **1**, exerting a mixed inhibition mode. These results and the low toxicity on human fibroblasts and on melanoma cells identified a promising lead molecule in compound **7** for the development of new whitener additives to therapeutic or cosmetics agents.

## Figures and Tables

**Table 1:** V_MAX_ (expressed in nA) and K_M_ (expressed as mM concentrations) obtained from biosensors’ calibrations, in absence or not of the inhibitor. For each inhibitor was built a group of biosensors (n = 4) and parameters’ variations were listed. Statistical significance (* *p* < 0.05 vs. dopamine) was evaluated by means of *t*-test. Type of inhibition and calculated IC_50_ were tabulated too. Concentrations were expressed in nM.

Compound	Dopamine		Inhibitor + Dopamine			
	V_MAX_ (nA)	K_M_ (mM)	V_MAX_ (nA)	K_M_ (mM)	Type of Inhibition	IC_50_ (nM)
**1**	157.4 ± 17.7	0.453 ± 0.221	143.6 ± 18.7	19.910 ± 7.610 *	COMPETITIVE	24
**6**	47.71 ± 1.6	1.348 ± 0.217	30.33 ± 2.8	10.320 ± 3.338 *	COMPETITIVE	11
**7**	256.7 ± 16.2	4.727 ± 1.074	185.9 ± 13.6 *	12.131 ± 2.763 *	MIXED	12

## Data Availability

Not applicable.

## Supplementary Materials

The following supporting information can be downloaded at: https://www.mdpi.com/article/10.3390/molecules27227942/s1: Figure S1: (Left) UV-Vis spectra of L-Tyr (2 mM) in 20 mM phosphate buffer, pH 7.4, pre-mix and after mix with the tyrosinase solution (0.12 mg/mL) at different time of incubation (indicated). (Right) Time-course of tyrosine oxidation by the tyrosinase/O_2_ oxidizing system. Data are the average of three replicates and the error is less than 5%; Figure S2: (Left) UV-Vis spectra of dopamine (2 mM) in 20 mM phosphate buffer, pH 7.4, pre-mix and after mix with the tyrosinase solution (0.12 mg/mL) at different time of incubation (indicated). (Right) Time-course of dopamine oxidation by the tyrosinase/O_2_ oxidizing system. Data are the average of three replicates and the error is less than 5%; Figure S3: cytotoxic activity of arbutin: cells were cultured with increasing concentrations (100, 200, 400 µM) of arbutin up to 48 h. Cell proliferation values were calculated as the growth percentages of treated cells compared to the untreated ones (CTR). The graph represents the results of three experiments, each done in triplicate; Figure S4: effects on tyrosinase-based biosensors (n = 4) of the exposition in a range comprised between 0 and 150 mM of dopamine, in absence (black line) and in presence (red line) of inhibitor 1. In Panel A, the Michaelis-Menten plots, showing the variations of both VMAX and KM, are reported, while in Panel B the relative Lineweaver–Burk plot is shown (all data are shown in Table 1); Figure S5: effects on tyrosinase-based biosensors (n = 4) of the exposition in a range comprised between 0 and 150 mM of dopamine, in absence (black line) and in presence (red line) of inhibitor 6. In Panel A, the Michaelis-Menten plots, showing the variations of both VMAX and KM, are reported, while in Panel B the relative Lineweaver–Burk plot is shown (all data are shown in Table 1); Figure S6: effects on tyrosinase-based biosensors (n = 4) of the exposition in a range comprised between 0 and 150 mM of dopamine, in absence (black line) and in presence (red line) of inhibitor 7. In Panel A, the Michaelis-Menten plots, showing the variations of both VMAX and KM, are reported, while in Panel B the relative Lineweaver–Burk plot is shown (all data are shown in Table 1); Figure S7: column graphs describing the effect of different concentrations of compound 1 (panel A), 6 (panel B) and 7 (panel C), ranging from 1 up to 40 µM, on viability of PC12 cells. *p* < 0.05 vs. control; Figure S8: representation of the most populated pose of arbutin surrounding the catalytic site with the most representative amino acids residues; Figure S9: representation of the most populated pose of 1 surrounding the catalytic site with the most representative amino acids residues; Figure S10: schematic representation of Tyrosinase-based biosensor used in this study, with tyrosinase reaction mechanism; Figure S11: schematic representation of platinum-based sensor used in this study to investigate the antioxidant proprieties of compound 1, 6 and 7. Pt/Ir: Platinum/Iridium (90:10) wire (1 mm in length; 125 µm in Ø); PPD: poly-ortho-phenylenediamine polymer; Table S1: docking list of ligands with amino acids of catalytic site of *B. megaterium* tyrosinase protein (3NM8); Table S2: H-bonds list of ligands with amino acids of catalytic site of *B. megaterium* tyrosinase protein (3NM8).

## Sample Availability

Samples of the compounds **1**, **6** and **7** are available from the authors.
