# Supplementary material for: Inhibitory Effect of Curcumin-Inspired Derivatives on Tyrosinase Activity and Melanogenesis
Source: Molecules. 2022 Nov 16;27(22):7942. doi: 10.3390/molecules27227942 (PMC9695798; doi:10.3390/molecules27227942)
Supplement: Supplementary file 1 [file molecules-27-07942-s001.zip › molecules-1997294-supplementary.pdf]

Supplementary Materials

Inhibitory Effect of Curcumin-Inspired Derivatives on Tyrosinase Activity and Melanogenesis

Gaia Rocchitta [1,†], Carla Rozzo [2,†], Marina Pisano [2,†], Davide Fabbri [3,*], Maria Antonietta Dettori [3,*], Paolo Ruzza [4], Claudia Honisch [4], Roberto Dallocchio [3], Alessandro Dessì [3], Rossana Migheli [1], PierAndrea Serra [1] and Giovanna Delogu [3]

[1] Dipartimento di Medicina, Chirurgia e Farmacia, Università degli Studi di Sassari, 07100 Sassari, Italy
[2] Istituto di Ricerca Genetica e Biomedica, Consiglio Nazionale Ricerche, 07100 Sassari, Italy
[3] Istituto di Chimica Biomolecolare, Consiglio Nazionale Ricerche, 07100 Sassari, Italy
[4] Istituto di Chimica Biomolecolare, Consiglio Nazionale Ricerche, 35131 Padova, Italy

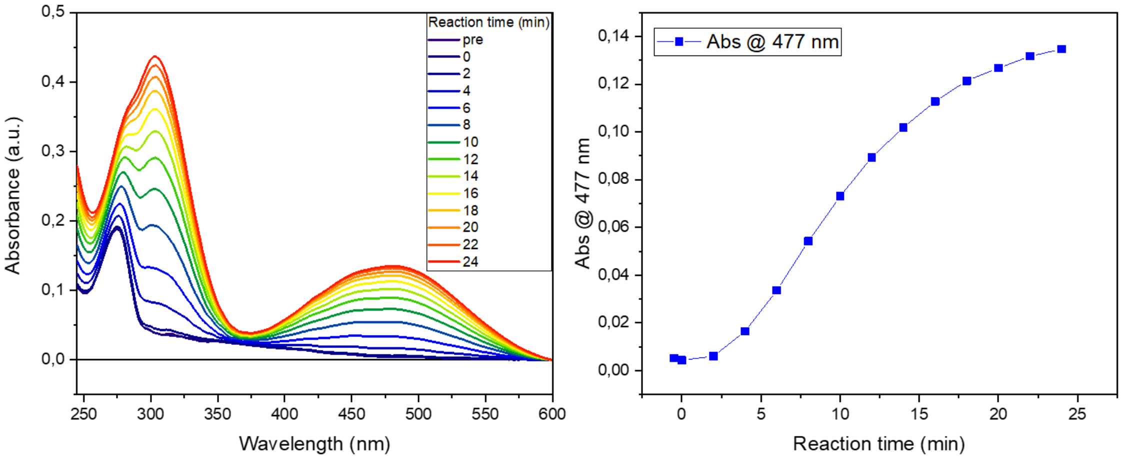

Figure S1. (**Left**) UV-Vis spectra of L-Tyr (2 mM) in 20 mM phosphate buffer, pH 7.4, pre-mix and after mix with the tyrosinase solution (0.12 mg/mL) at different time of incubation (indicated). (**Right**) Time-course of tyrosine oxidation by the tyrosinase/O2 oxidizing system. Data are the average of three replicates and the error is less than 5%.

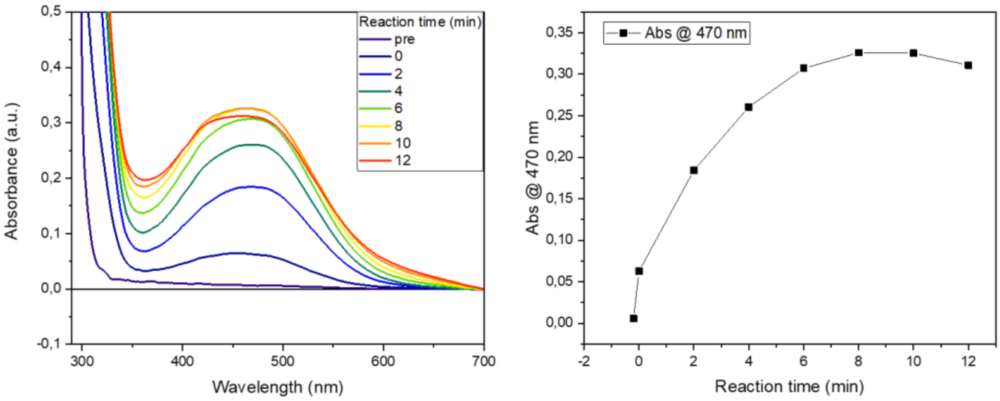

Figure S2. (**Left**) UV-Vis spectra of dopamine (2 mM) in 20 mM phosphate buffer, pH 7.4, pre-mix and after mix with the tyrosinase solution (0.12 mg/mL) at different time of incubation (indicated). (**Right**) Time-course of dopamine oxidation by the tyrosinase/O2 oxidizing system. Data are the average of three replicates and the error is less than 5%.

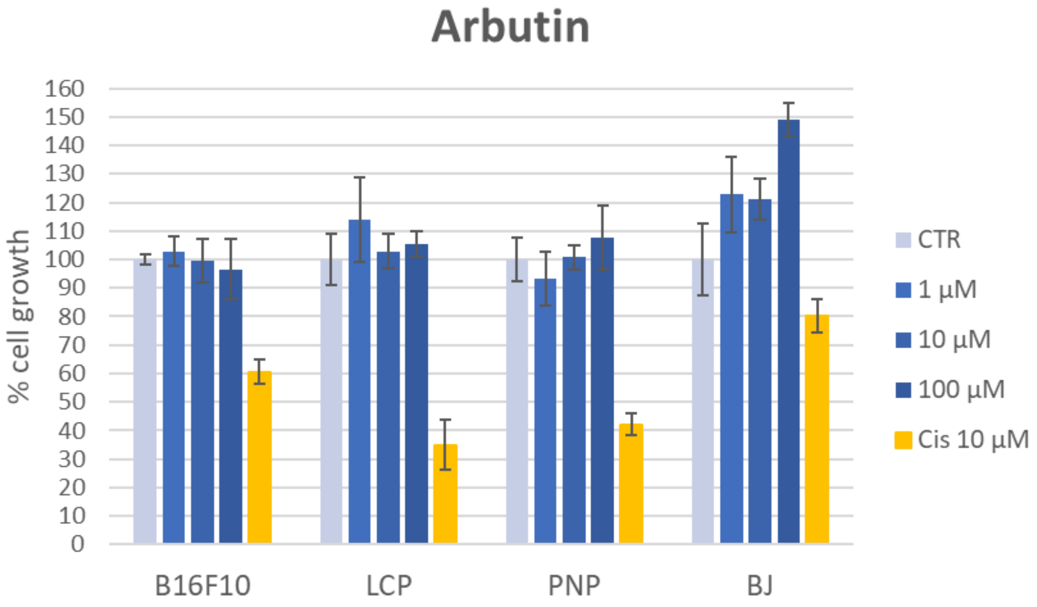

Figure S3. Cytotoxic activity of arbutin: cells were cultured with increasing concentrations (1, 10, 100 µM) of arbutin and cisplatin (10 µM) up to 48 h. Cell proliferation values were calculated as the growth percentages of treated cells compared to the untreated ones (CTR). The graph represents the results of three experiments, each done in triplicate.

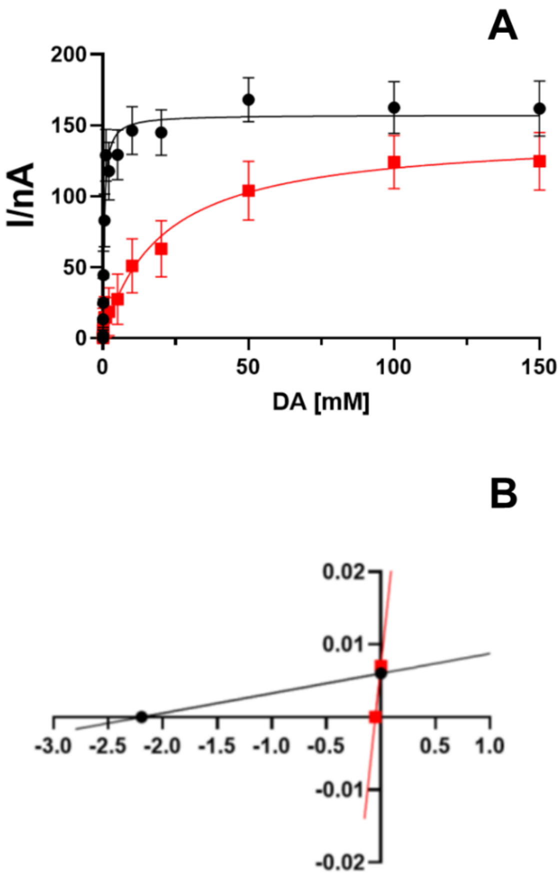

Figure S4. Effects on tyrosinase-based biosensors (n=4) of the exposition in a range comprised between 0 and 150 mM of dopamine, in absence (black line) and in presence (red line) of inhibitor 1.

In Panel A, the Michaelis-Menten plots, showing the variations of both V_{MAX} and K_M, are reported, while in Panel B the relative Lineweaver–Burk plot is shown (all data are shown in Table S1).

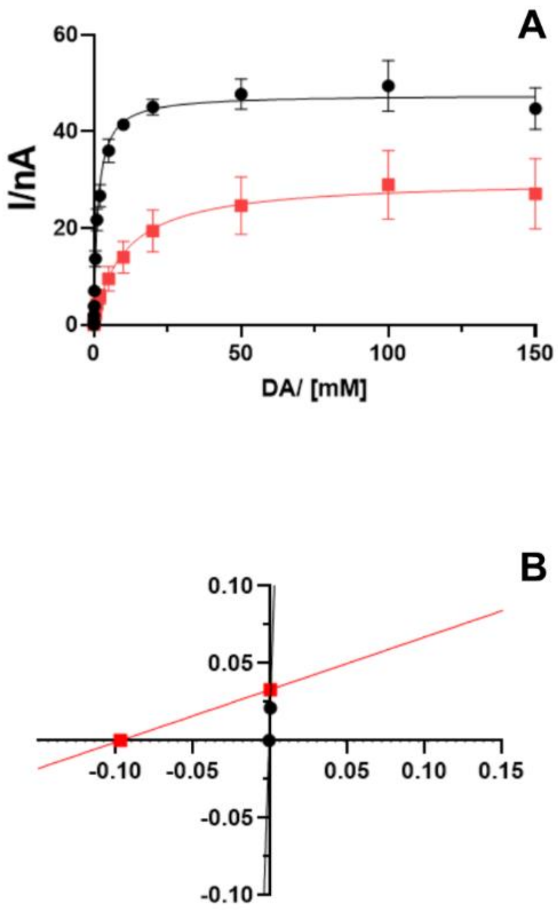

Figure S5. Effects on tyrosinase-based biosensors (n=4) of the exposition in a range comprised between 0 and 150 mM of dopamine, in absence (black line) and in presence (red line) of inhibitor **6**. In Panel A, the Michaelis-Menten plots, showing the variations of both V_{MAX} and K_M, are reported, while in Panel B the relative Lineweaver–Burk plot is shown (all data are shown in Table S1).

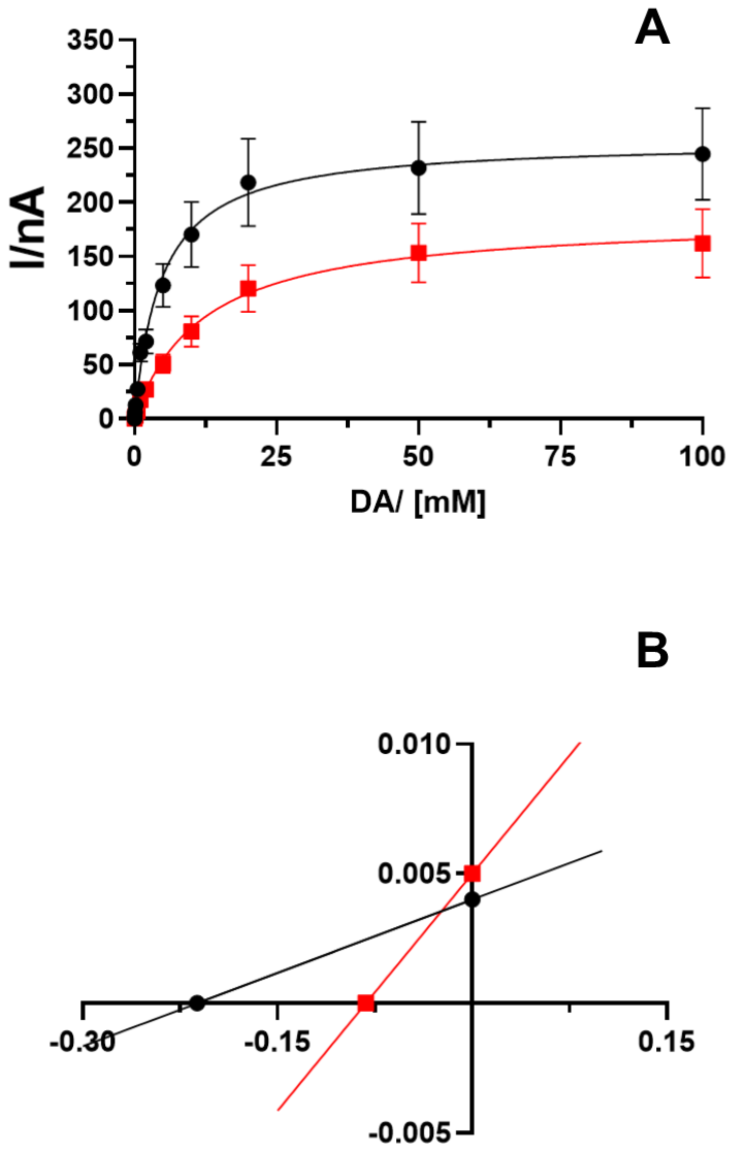

Figure S6. Effects on tyrosinase-based biosensors (n=4) of the exposition in a range comprised between 0 and 150 mM of dopamine, in absence (black line) and in presence (red line) of inhibitor **7**. In Panel A, the Michaelis-Menten plots, showing the variations of both V_{MAX} and K_M, are reported, while in Panel B the relative Lineweaver–Burk plot is shown (all data are shown in Table S1).

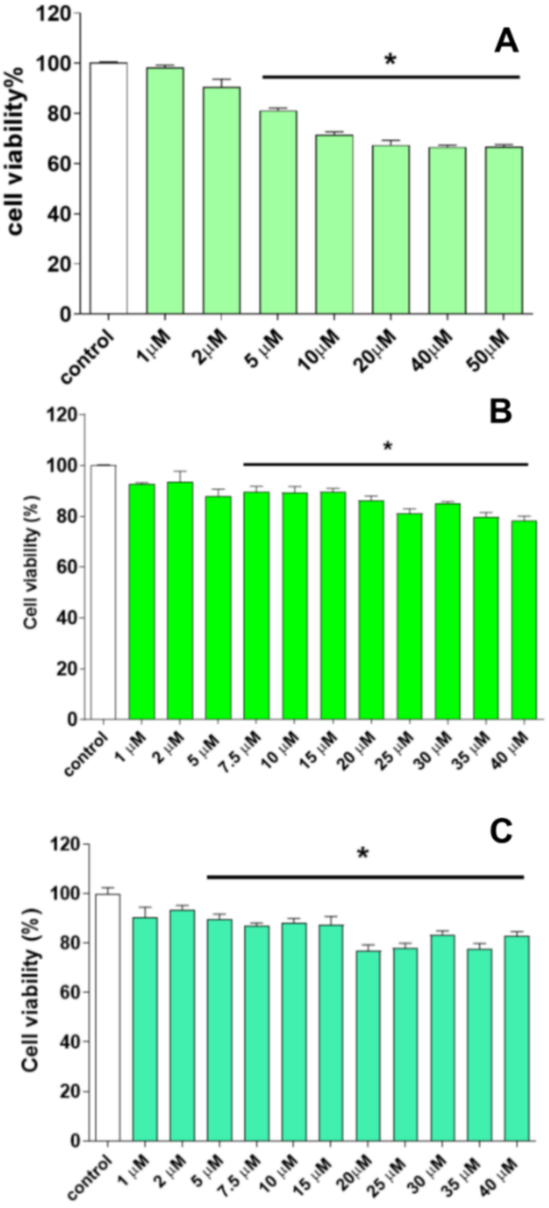

Figure S7. Column graphs describing the effect of different concentrations of compound **1** (panel A), **6** (panel B) and **7** (panel C), ranging from 1 up to 40 μM, on viability of PC12 cells. $p<0.05$ vs control.

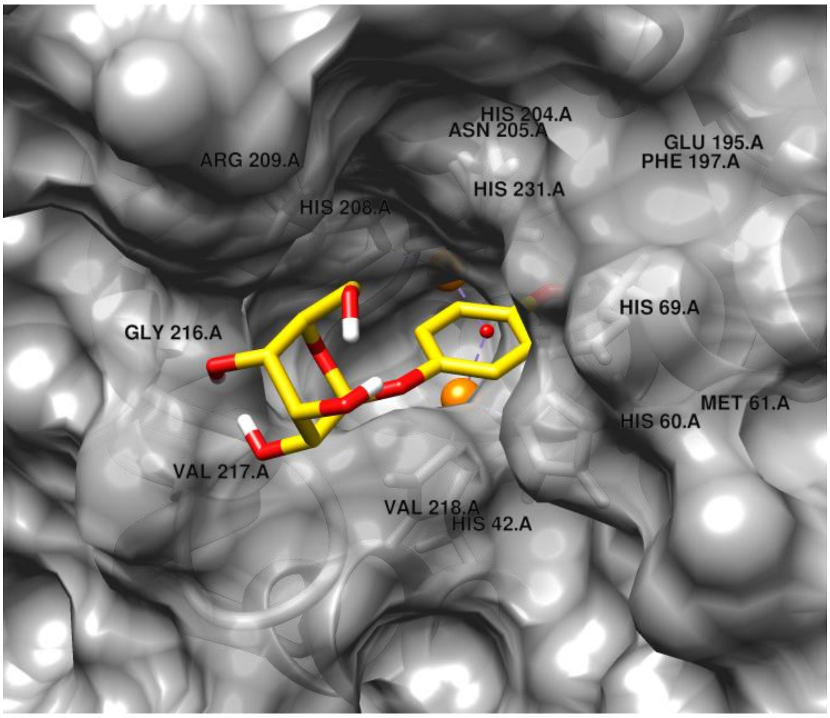

Figure S8. Representation of the most populated pose of arbutin surrounding the catalytic site with the most representative amino acids residues.

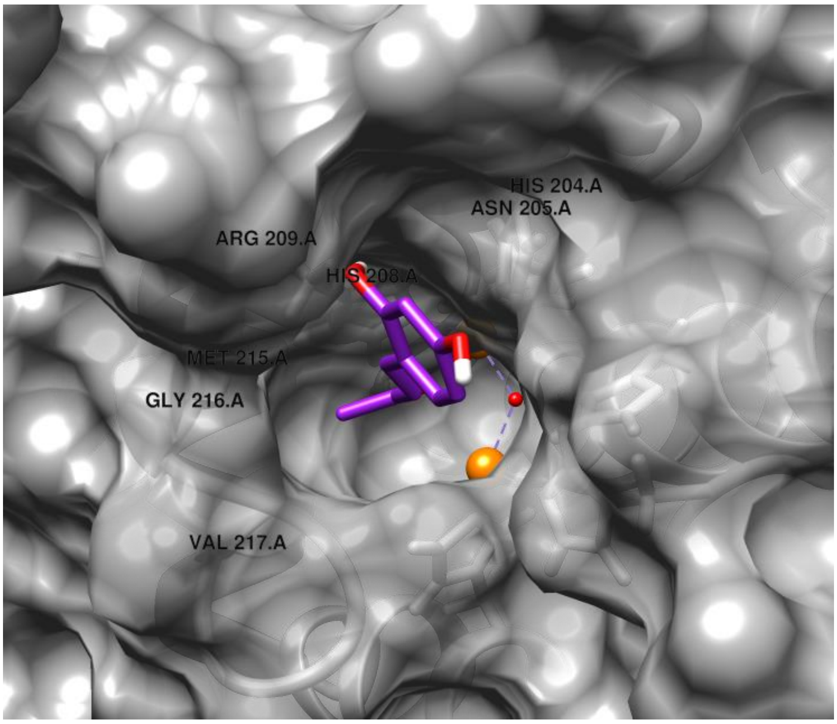

Figure S9. Representation of the most populated pose of **1** surrounding the catalytic site with the most representative amino acids residues.

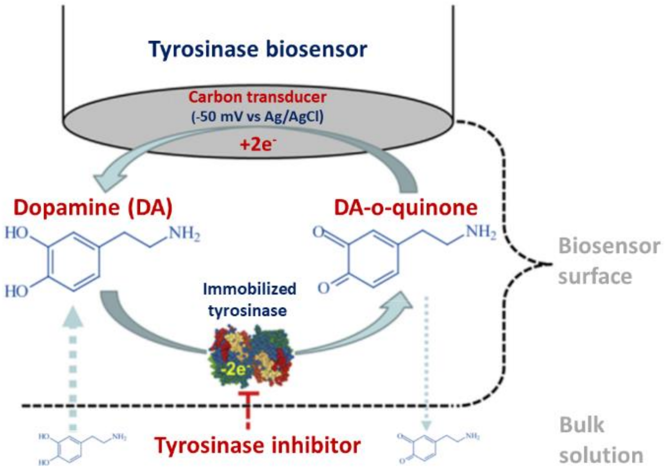

Figure S10. Schematic representation of Tyrosinase-based biosensor used in this study, with tyrosinase reaction mechanism.

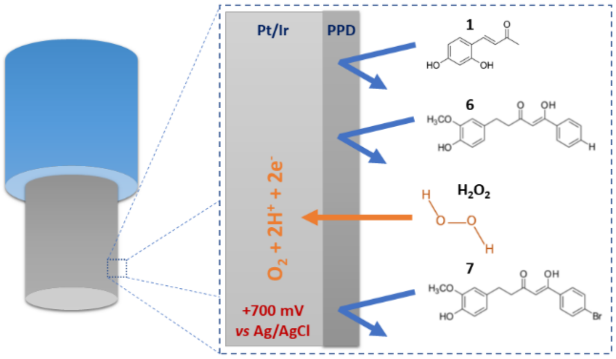

Figure S11. Schematic representation of Platinum-based sensor used in this study to investigate the antioxidant proprieties of compound **1**, **6** and **7**. Pt/Ir: Platinum/Iridium (90:10) wire (1 mm in length; 125 µm in Ø); PPD: poly-ortho-phenylenediamine polymer.

Table S1. Docking list of ligands with amino acids of catalytic site of *B. megaterium* tyrosinase protein (3NM8): [a]M.B.E.: Mean Binding Energy, [b]E.F.E.B.: Estimated Free Energy of Binding, [c]E.I.C.: Estimated Inhibition Constant, Ki.

	In ligands	%	M.B.E.[a]	E.F.E.B.[b]	E.I.C., Ki[c]	Interactions, **HBond**
1	**KojicAcid**.1.c13.r51	13	-4.22	-4.23	789.57 uM	His60 **Glu195** His204 **Asn205** His208 **Gly216** Val218
	**KojicAcid**.2.c60.r16	60	-3.70	-4.00	1.17 mM	His204 **Asn205** His208 **Arg209** Gly216
	**KojicAcid**.3.c12.r100	12	-3.51	-3.56	2.47 mM	**Glu158 Phe197 Gly200** Pro201 **Arg209**
	**KojicAcid**.4.c8.r91	8	-3.49	-3.50	2.72 mM	**Asn205** His208 **Arg209** Gly216 Val217 **Val218**
2	**6**.1.c10.r70	10	-6.29	-6.97	7.82 uM	His60 **Glu158** Glu195 Phe197 Gly200 Pro201 His204 Asn205 His208 **Arg209**
	**6**.4.c18.r97	18	-5.19	-5.82	53.99 uM	Gly200 Pro201 **Asn205** His208 **Arg209** Gly216 Val217 **Val218** Pro219

**3**	**7**.1.c6.r85	6	-6.60	-7.05	6.79 uM	His60 Met61 Phe197 Gly200 Pro201 His204 **Asn205** His208 **Arg209** Gly216 Val217 Val218
	**7**.3.c18.r98	18	-5.94	-6.61	14.37 uM	Phe197 Gly200 Pro201 **Asn205** His208 **Arg209** Gly216 Val217 **Val218** Pro219
4	**1**.1.c33.r78	33	-5.27	-5.37	116.31 uM	**Glu158** Phe197 Gly200 Pro201 Asn205 **Arg209**
	**1**.3.c39.r50	39	-5.07	-5.12	176.56 uM	His204 **Asn205** His208 **Arg209** Met215 Gly216 Val217
	Arbutin.1.c9.r71	9	-3.33	-3.91	1.35 mM	**Glu158** Phe197 Gly200 Pro201 Asn205 **Arg209**
**5**	Arbutin.2.c50.r78	50	-3.10	-3.62	2.23 mM	His60 Met61 **Glu195** Phe197 His204 **Asn205** Arg209 **Gly216** Val217 Val218
	Arbutin.4.c15.r99	15	-3.14	-3.32	3.66 mM	**Glu158** Phe197 Gly200 Pro201 Asn205 **Arg209** **Gly216** Val218

Table S2. H-bonds list of ligands with amino acids of catalytic site of *B. megaterium* Tyrosinase protein (3NM8).

				**Hydrogen bond interactions**			
**pose**	**Tested Ligands**	**%**	**H-bond**	**Ligand Atom**	**Protein Atom**	**Distance (Å)[c]**	**Ang. (°)[c]**
1	**Kojic Acid**	13	3	H10(HD)	Glu195:OE1(OA)	2.000	162.49
				O9(OA)	Asn205:1HD2(HD)	1.855	147.85
				H12(HD)	Gly216:O(OA)	2.212	111.59
2		60	2	H10(HD)	Asn205:O(OA)	2.129	137.75
				O9(OA)	Arg209:2HH1(HD)	2.152	162.22
3		12	4	H10(HD)	Glu158:OE2:(OA)	2.075	111.96
				H12(HD)	Phe197:O(OA)	2.061	150.81
				O11(OA)	Gly200:HN(HD)	2.416	135.33
				O9(OA)	Arg209:1HH1(HD)	1.862	168.53
4		8	3	H12(HD)	Asn205:O(OA)	1.770	136.66
				O7(OA)	Arg209:2HH1(HD)	2.116	162.37
				O9(OA)	Val218:HN(HD)	2.062	165.48
1	**6**	10	4	H12(HD)	Glu158:OE2:(OA)	2.295	158.77
				O14(OA)	Arg209:1HH1(HD)	**2.592**	**80.45**
				O14(OA)	Arg209:2HH1(HD)	**2.005**	**116.48**
				O17(OA)	Arg209:1HH1(HD)	**1.706**	**156.86**
4		18	3	H18(HD)	Asn205:O(OA)	2.276	155.33
				O17(OA)	Arg209:2HH1(HD)	2.072	166.16
				O11(OA)	Val218:HN(HD)	2.038	166.81
1	**7**	6	2	H18(HD)	Asn205:O(OA)	2.421	114.27
				O17(OA)	Arg209:2HH1(HD)	2.102	156.62
3		18	4	O2(OA)	Asn205:2HD2(HD)	**2.557**	**102.25**
				H18(HD)	Asn205:O(OA)	**2.020**	**138.08**
				O17(OA)	Arg209:2HH1(HD)	2.036	160.25
				O13(OA)	Val218:HN(HD)	2.029	144.83
1	**1**	33	3	H13(HD)	Glu158:OE2:(OA)	1.798	149.08

				O10(OA)	Arg209:2HH1(HD)	**1.993**	**165.41**
				O12(OA)	Arg209:2HH1(HD)	**1.775**	**174.29**
3		39	2	H13(HD)	Asn205:O(OA)	2.086	121.21
				O12(OA)	Arg209:2HH1(HD)	2.209	173.60
1		9	4	H20(HD)	Glu158:OE2:(OA)	**2.258**	**108.67**
				H22(HD)	Glu158:OE2:(OA)	**2.091**	**176.80**
				O19(OA)	Arg209:1HH1(HD)	**2.373**	**119.36**
				O21(OA)	Arg209:1HH1(HD)	**1.728**	**161.80**
2	**Arbutin**	50	4	H15(HD)	Glu195:OE1(OA)	1.806	170.45
				O14(OA)	Asn205:1HD2(HD)	2.229	145.32
				H17(HD)	Gly216:O(OA)	**2.149**	**172.64**
				H24(HD)	Gly216:O(OA)	**1.827**	**149.22**
4		15	4	H15(HD)	Glu158:OE2:(OA)	2.126	162.34
				O14(OA)	Arg209:1HH1(HD)	2.466	166.05
				H17(HD)	Gly216:O(OA)	**1.686**	**149.18**
				H24(HD)	Gly216:O(OA)	**2.245**	**111.94**

[a]Oxygen acceptor, [b]Hydrogen donor, [c]Cross-bridge H-bond interactions with the same aa are listed in bold.
